# A Fine-Tuned Large Language Model Workflow to Support Risk of Bias Assessment: Development, External Validation, and Reviewer Verification Study

**DOI:** 10.2196/108001

**Published:** 2026-11-13

**Authors:** Yitian Li, Jingyu Chen, Liting Liang, Xingyun Geng, Guohua Wang, Jinsong Geng

**Affiliations:** 1 Department of Medical Informatics, Medical School of Nantong University, Nantong, Jiangsu, China; 2 Center for Evidence-Based Medicine, Medical School of Nantong University, 19 Qixiu Road, Medical School of Nantong University, Nantong, Jiangsu, 226001, China, 86 51385051891; 3 National Stroke Administration Office, Xuanwu Hospital Capital Medical University, Beijing, China

**Keywords:** large language model, risk of bias, randomized controlled trial, workflow, validation

## Abstract

**Background:**

Given that randomized controlled trials (RCTs) are the gold standard for effectiveness research, assessing their risk of bias (RoB) becomes an indispensable step in systematic reviews. Yet this task remains time-consuming and prone to reviewer subjectivity. While large language models may accelerate the process, they lack transparent, traceable judgments linked to trial reports.

**Objective:**

To bridge methodological gaps and improve both efficiency and consistency, this study developed and externally validated TRoB-1, a fine-tuned open-weight large language model workflow for RoB assessment of RCTs.

**Methods:**

TRoB-1 was developed specifically for the Cochrane risk of bias tool version 1 (RoB 1), which is the most widely adopted instrument for critical appraisal of RCTs. The workflow generates preliminary judgments and supporting quotations from reviewer-selected passages for the target RoB domains. The workflow was built on 2-stage supervised fine-tuning of Gemma 2-9B-Instruct using 4-bit quantized low-rank adaptation. The training set comprised 4321 RoB records extracted from systematic reviews of chronic obstructive pulmonary disease, diabetes, and hypertension. The validation set consisted of 161 records from recently published RCTs covering a broader disease area. The reference standard was established by 2 independent reviewers. Two clinicians blinded to the reference standard verified TRoB-1-assisted assessments through a locally hosted web interface. Using the same reference standard, the performance of TRoB-1 raw outputs was compared with that of the Gemma 2 base workflow, and clinician-verified outputs were compared with raw TRoB-1 outputs.

**Results:**

TRoB-1 successfully generated structured outputs for all 161 validation records. In independent clinician-assisted assessments using the same workflow, raw TRoB-1 accuracy ranged from 73.3% (n=118) to 73.9% (n=119), compared with 52.2% (n=84) to 60.2% (n=97) for the Gemma 2 base model. After verification, accuracy ranged from 82.0% (n=132) to 84.5% (n=136) and macro-*F*_1_ from 0.709 to 0.776. Accuracy remained higher for raw TRoB-1 after excluding invalid base-model outputs, and verification did not change any correct judgment to an incorrect one. Clinician 1 corrected 13 initially incorrect judgments and clinician 2 corrected 18. The *F*_1_-score for judgments of high-RoB increased from 0.600 to 0.784 for clinician 1 and from 0.571 to 0.821 for clinician 2.

**Conclusions:**

TRoB-1 combined task-specific fine-tuning, evidence-linked outputs, and clinician verification in a transparent workflow for reviewer-supported RoB assessment. The TRoB-1 web interface provides a practical platform for integrating RoB into evidence synthesis, particularly for living systematic reviews where timely updates are critical.

## Introduction

Randomized controlled trials (RCTs) are regarded as the gold-standard of primary evidence for assessing the comparative effectiveness of therapeutic interventions. RCTs randomly assign participants to either the intervention group or the control group. They serve as a major source of evidence for conducting systematic reviews and formulating clinical practice guidelines, thereby supporting evidence-informed decision-making [1,2]. However, not all RCTs have trustworthy findings. A high-quality trial depends on rigorous design, proper implementation, and transparent reporting. Any methodological flaws will undermine the credibility of the evidence, and these flaws can be detected through risk of bias (RoB) assessment.

The Cochrane risk-of-bias tool version 1 (RoB 1) is the most widely adopted instrument for critical appraisal of RCTs [3]. The validated tool enables systematic assessment of potential biases. Although the new version of the Cochrane RoB tool is more comprehensive, it yields low interrater reliability and poses challenges in its application even for experienced raters in systematic reviews [4]. Researchers frequently misinterpret assessment rules and inaccurately judge domain-specific bias, making the assessment highly time-consuming and resource-intensive [5,6]. Therefore, the majority of non-Cochrane reviews published still continue to adopt the conventional RoB 1 tool [7].

To alleviate the heavy workload and inconsistent judgments, automated RoB assessment has been increasingly explored to streamline the procedure [8-10]. Previous text-mining and machine learning systems have shown that computational algorithms can extract bias-related text and generate domain-specific judgments. However, such systems only produce domain-level labels, prefilled RoB fields, or highlighted text. None of these tools provide researchers with workflows that incorporate traceable quotations, task-specific explanations, and record-level verifications [10-12]. These disadvantages are critical as RoB assessment requires not only judgments but also reliable justifications.

In recent years, large language models (LLMs) have emerged as a promising technical solution due to their capability to analyze texts, follow task-specific instructions, and generate structured outputs. LLMs have been deployed for conducting systematic reviews, such as literature screening, data extraction, and evidence synthesis [13]. Although general-purpose LLMs can produce seemingly plausible responses, they may yield inaccurate statements or inconsistent judgments [14].

Despite these explorations, substantial challenges persist in LLM-assisted appraisal practice. Most studies adopted proprietary models accessible through web interfaces or application programming interfaces, an approach that limits study reproducibility and data governance. In addition, RoB assessment in prior studies mainly depended on prompting instead of task-specific adaptation. Parameter-efficient approaches, like low-rank adaptation and quantized low-rank adaptation (QLoRA), would reduce the computational resources needed for local fine-tuning [15,16]. Moreover, a deployable RoB assessment platform should be characterized as a workflow rather than a classifier alone [17].

To address the methodological gaps in LLM-assisted RoB assessment, our study aimed to develop and externally validate TRoB-1, a fine-tuned open-weight LLM workflow that enables RoB appraisal for RCTs and improves the transparency and traceability of the appraisal process.

## Methods

### Study Design

This study was reported in accordance with the TRIPOD-LLM (Transparent Reporting of a Multivariable Model for Individual Prognosis or Diagnosis–Large Language Model) reporting guideline for studies using LLMs (Checklist 1) [18]. The study workflow comprised model development, reference standard construction, external validation, and reviewer verification (Figure 1). For each assessment record, TRoB-1 generated a preliminary RoB 1 judgment from source passages selected by a reviewer. The unit of analysis was the RoB assessment record, defined as a study-domain assessment or, when applicable, a study-outcome-domain assessment. The training dataset contained 4321 assessment records.

**Figure 1.**
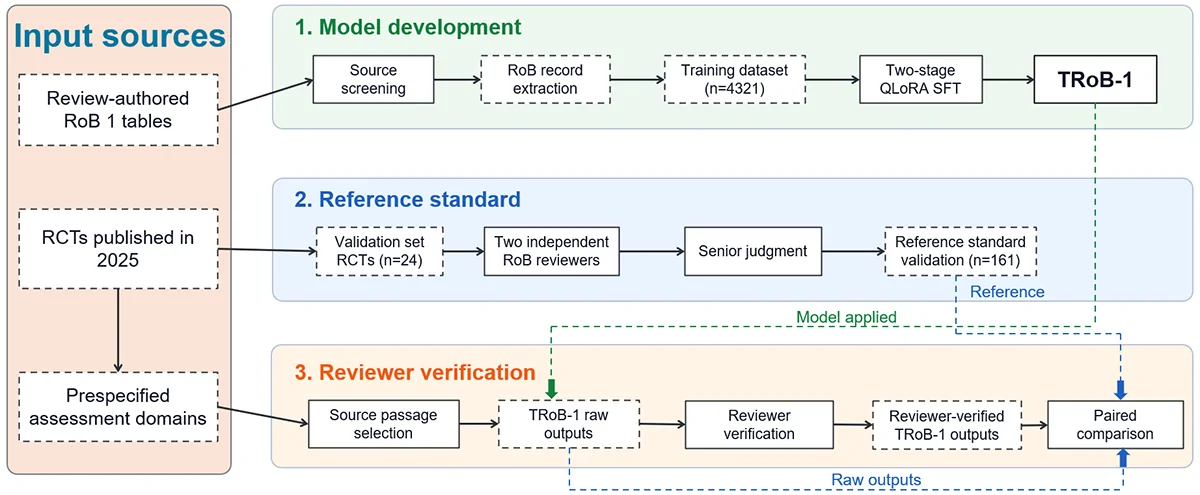
Study workflow for TRoB-1 development, reference-standard construction, and reviewer verification. QLoRA: quantized low-rank adaptation; RCT: randomized controlled trial; RoB: risk of bias; RoB 1: Cochrane risk-of-bias tool version 1; SFT: supervised fine-tuning.

### Domains of RoB Assessment

We used RoB 1, the Cochrane RoB tool for RCTs, which serves as the predominant instrument in systematic reviews to assess the quality of the included trials. The workflow for our RoB assessment covered 6 domains: random sequence generation, allocation concealment, blinding of participants and personnel, blinding of outcome assessment, incomplete outcome data, and other sources of bias. Selective reporting was handled in a separate manual assessment module, as its reliable judgment requires comparison across trial reports, protocols, registries, and prespecified outcomes, which were beyond the scope of our available data. Furthermore, even in Cochrane reviews with mandated protocols, the majority of judgments on this domain have been found to be erroneous [19]. Judgments of bias in our study were classified as “low risk,” “unclear risk,” or “high risk.” Outcome-dependent domains were assessed at the outcome level, with outcomes categorized as subjective, objective, or both [20].

### Model Training

Training data were derived from systematic reviews and meta-analyses on chronic obstructive pulmonary disease (COPD), diabetes, and hypertension. A literature search was carried out in PubMed. Search terms involved MeSH terms and keywords of the diseases. Systematic reviews and meta-analyses published between January 2015 and November 2025 were identified. Eligible reviews had to use RoB 1 to assess the included RCTs, while reviews that used other bias assessment tools were excluded.

Each RCT contributed one record for each of the RoB 1 domains: random sequence generation, allocation concealment, blinding of participants and personnel, incomplete outcome data, and other bias. Blinding of outcome assessment was assessed separately for each applicable outcome type, yielding one additional record per outcome type.

Records were kept separate by domain, evidence, and outcome when applicable. One reviewer performed initial screening and deduplication. Records that remained with conflicting judgments for the same trial and domain were resolved by consensus with a second reviewer. The final dataset was jointly checked by both reviewers. It comprised 4321 RoB 1 assessment records from 199 systematic reviews, corresponding to 1753 unique RCTs.

All training data were formatted following the ShareGPT conversational format. The RoB judgments and supporting text were then converted into instruction-following examples to facilitate supervised fine-tuning. The training input included the RoB 1 domain, supporting text, and outcome type when applicable. Candidate evidence hints were not used for model training. Instead, they were automatically generated by predefined rules prior to Step 1. While the supervised output was a structured JSON object containing the bias judgment and a quotation extracted from the text.

TRoB-1 was developed from Gemma 2-9B-Instruct (Google) through 2-stage supervised fine-tuning using QLoRA. The first stage adapted the model to make RoB judgment. The second stage focused on improving its adherence to the required structured output format. The training environment and parameters used in the fine-tuning stages are shown in Multimedia Appendix 1.

### Construction of an External Validation Set and Reference Standard

We searched PubMed on January 13, 2026, to identify RCTs published in 2025 on COPD, diabetes, hypertension, and additional clinical areas, including oncology, neurosurgery, dermatology, rheumatology, obstetrics, and hepatology. The literature was screened according to the prespecified eligibility criteria.

Eligible RCTs were grouped by clinical area, and each was assigned a unique identification number. Random sampling was then performed independently within each group using a random number table. To test the applicability of TRoB-1 judgments across various clinical contexts, 6 RCTs were selected from each of the COPD, diabetes, and hypertension groups, and 1 RCT was selected from each of the other 6 clinical areas. The final validation set therefore comprised 24 RCTs. We checked all included RCTs by title, DOI, and registry identifier where available and grouped publications of the same trial together. No overlap of trials was found between the validation and training sets. For trials without a registry identifier, we additionally used report titles, DOIs, author names, trial settings, and types of interventions and comparisons (Multimedia Appendix 2).

Two reviewers experienced in RoB assessment independently assessed each record. Both reviewers held MD and PhD degrees and had more than 10 years’ experience in teaching evidence-based medicine. Both of them had published Cochrane reviews and other systematic reviews. Before the formal assessment, they reviewed the RoB 1 guidance and agreed on the assessment criteria. They were permitted to consult trial protocols and registry records.

Before adjudication, the 2 reviewers agreed on 149 of 161 records, with 12 disagreements across 8 RCTs. Disagreements occurred in blinding of participants and personnel (n=2), blinding of outcome assessment (n=5), incomplete outcome data (n=2), and other sources of bias (n=3). Disagreements were first discussed between the 2 reviewers. Unresolved cases were referred to a senior expert and resolved by consensus. The final consensus served as the reference standard, which was used to evaluate the performance of the Gemma 2 base workflow, TRoB-1 raw outputs, and clinician-verified TRoB-1 outputs.

### TRoB-1 Inference and Output Verification

The TRoB-1 assessment workflow comprised 2 sequential steps. Before assessment, we provided the reviewers with the RoB assessment guidance according to the Cochrane Handbook for Systematic Reviews of Interventions. For each assessment, the reviewer first selected the target RoB domain and, when applicable, the outcome type, and then selected the source passage from the relevant section according to the section headings. These reviewer-selected passages served as the input to TRoB-1. The selected outcome type was included as optional context in the model input.

Prior to Step 1, a deterministic, domain-specific rule-based procedure generated up to 3 optional candidate evidence hints from the selected passages. These hints only prioritized potentially relevant sentences within the passages. The detailed generation procedure, rules, and distributions are provided in Multimedia Appendix 3.

In Step 1, TRoB-1 generated a RoB judgment and an exact supporting quotation in JSON format. In Step 2, TRoB-1 used the judgment and supporting quotation from Step 1, together with the corresponding domain-specific RoB 1 rule summary, to generate a brief explanation. The domain-specific rule summaries are provided in Multimedia Appendix 4.

Outputs in Step 1 were checked automatically for completeness, use of an accepted judgment category, and traceability of the supporting quotation. Outputs that failed any check were regenerated using the predefined retry procedure. The TRoB-1 raw outputs were defined as the model’s final outputs after retry and before reviewer verification. Each judgment and supporting quotation can be revised when necessary. The final outputs were defined as clinician-verified assessments. The complete workflow for TRoB-1-assisted assessment is shown in Figure 2.

Two clinicians who had not participated in establishing the reference standard independently completed the TRoB-1-assisted workflow for all 161 records. For each prespecified RoB 1 domain, each clinician selected the source passage, reviewed the TRoB-1 output, and finalized the assessment. They were blinded to the reference standard and to each other’s selections, outputs, and judgments. For each record, both clinicians viewed the preliminary judgment, the supporting quotation, and the Step 2 explanation before finalizing the assessment. They were instructed to check each of these outputs against the source evidence and the RoB 1 criteria, revise it when necessary, and take responsibility for the final judgment. Prompt templates and expected outputs are provided in Multimedia Appendix 5. Model loading and decoding settings as well as predefined rules for output checks are provided in Multimedia Appendix 6.

**Figure 2.**
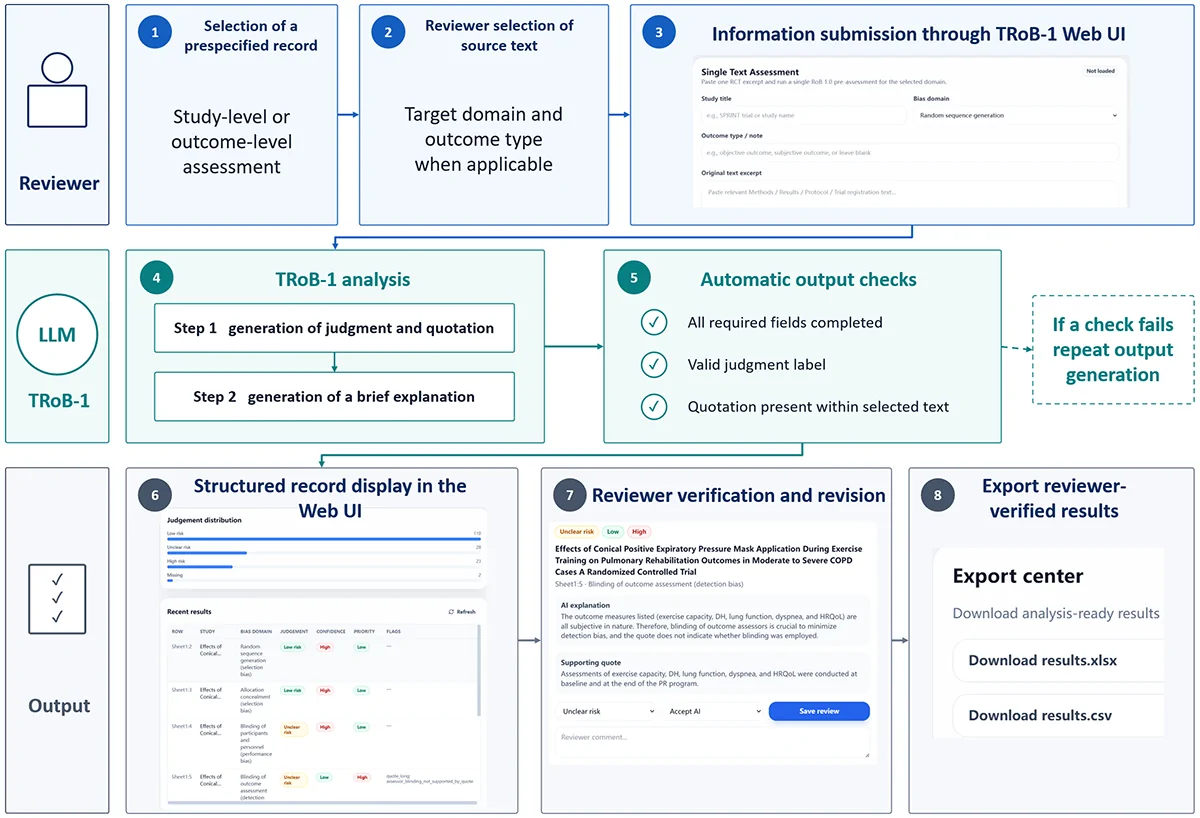
Web-based TRoB-1 assessment workflow. For each assessment record, the clinician selected the relevant source-text excerpt and specified the target RoB 1 domain and outcome type, when applicable. TRoB-1 generated a structured preliminary judgment and supporting quotation, applied predefined automated output checks, and produced a brief explanation. Two clinicians independently verified and, when necessary, revised the assessment before export. LLM: large language model; RoB 1: Cochrane risk-of-bias tool version 1; UI: user interface.

### Development of the TRoB-1 Web Interface

To support external validation, we deployed the TRoB-1 workflow through a locally hosted web interface. Assessment records were organized by project, allowing reviewers to perform single or batch assessments of RCTs, check and revise preliminary judgments, and export the records. Each record contained the preliminary judgment, supporting quotation, explanation, and any reviewer revisions or comments. The external-validation assessments were also conducted through this interface.

### Outcome Measures

The primary outcome was agreement between TRoB-1 raw judgments and the reference standard. Secondary outcomes included the proportion of valid automated outputs, class-specific performance, agreement after reviewer verification, and the proportion of judgments matching the reference standard before vs after verification. A valid output was a structured assessment record whose supporting quotation could be found in the passage selected by the clinician. It was available for checking and revision in the web interface and for subsequent export in CSV or Microsoft Excel Open XML Workbook (.xlsx) format. Judgment changes referred to whether the judgment category during retries differed from the initial output, and these changes were recorded separately from validity.

Time requirements were recorded for the construction of the reference standard and for the operation of the TRoB-1 workflow. For the TRoB-1 workflow, timing was measured according to supporting passage selection, model inference, and output verification. Agreement between the 2 clinicians’ final assessments was also analyzed.

### Statistical Analysis

Accuracy and macro-*F*_1_ were used as the main performance measures. Macro-*F*_1_ gives equal weight to the *F*_1_-score of each judgment category. Precision, recall, and *F*_1_-scores were calculated separately for judgments of low-risk, unclear-risk, and high-RoB. The Matthews correlation coefficient (MCC) was adopted to summarize overall classification performance across the 3 judgment categories [21]. Cohen κ and Gwet AC1 (Gwet first-order agreement coefficient) were used as chance-corrected agreement measures. Gwet AC1 was reported alongside Cohen κ because uneven category distributions can affect agreement estimates [22,23].

TRoB-1 raw outputs were compared with the Gemma 2 base workflow. Both workflows used the same validation records, supporting text, and inference settings. Invalid or incomplete base-model outputs were counted as incorrect in the primary analysis. Clinician-verified outputs were also compared against raw TRoB-1 outputs using the same reference standard. To investigate the robustness of the findings, we performed a sensitivity analysis restricted to the subset of records for which the base model successfully produced a valid structured output.

Performance metrics were calculated across the 161 assessment records independently by 2 clinicians. We estimated 95% CIs for overall metrics, paired differences, class-specific *F*_1_-scores, and domain- and validation-group–specific accuracy using 50,000 RCT-cluster bootstrap replicates. Paired accuracy comparisons used an exact paired permutation test at the RCT level. As a sensitivity analysis, accuracy was calculated within each RCT and averaged across the 24 RCTs with equal weight. Trial-level paired differences were evaluated using bootstrap CIs and an exact sign-flip test. Statistical analyses were performed using Python (version 3.11.6). Package versions are provided in Multimedia Appendix 7.

### Ethics Considerations

This study was based exclusively on publicly accessible data, including published peer-reviewed systematic reviews, RCTs, and public clinical trial registries. No individual patient data were collected or analyzed. The study was approved by the Medical Ethics Committee of Nantong University (ethical approval number 202303). As the study did not involve human participants or identifiable personal data, informed consent was not required.

## Results

### Overview

Fine-tuning led to a significant improvement in agreement with the reference standard compared to the Gemma 2 base workflow. Clinician verification also increased agreement in RoB assessments. None of the raw judgments that initially matched the reference standard became nonmatching after verification. Among the 3 judgment categories, judgments of high-RoB showed the largest class-specific increase in *F*_1_-score, rising from 0.600 to 0.784 for clinician 1 and from 0.571 to 0.821 for clinician 2. Notably, the TRoB-1 workflow generated well-formed structured outputs for all 161 validation records. The TRoB-1 interface presents RoB judgments and supporting quotations, along with modification options for researchers.

### Performance of External Validation

The external validation set consisted of 161 RoB assessment records across 6 domains. Selective reporting was assessed manually and was therefore excluded from the performance analyses. The reference standard comprised 110 low-risk, 24 unclear-risk, and 27 high-RoB records (Table 1).

Compared with the Gemma 2 base workflow, TRoB-1 raw outputs increased accuracy by 13.7% (95% CI 5.7%‐22.2%; *P*=.004) for clinician 1 and 21.1% (95% CI 11.7%‐30.4%; *P*<.001) for clinician 2. Macro-*F*_1_ and MCC were also higher (Table 2 and Figure 3). Detailed paired differences and their 95% CIs are shown in Multimedia Appendix 8.

**Table 1.** Characteristics of included randomized controlled trials (RCTs) and bias judgments in the external validation set.

Characteristics	Records, n	Low risk, n (%)	Unclear risk, n (%)	High risk, n (%)
Clinical area				
Chronic obstructive pulmonary disease	41	28 (68.3)	7 (17.1)	6 (14.6)
Diabetes	40	27 (67.5)	6 (15)	7 (17.5)
Hypertension	40	28 (70)	6 (15)	6 (15)
Out-of-domain	40	27 (67.5)	5 (12.5)	8 (20)
Total	161	110 (68.3)	24 (14.9)	27 (16.8)
RoB 1^a^ domain				
Random sequence generation (selection bias)	24	21 (87.5)	3 (12.5)	0 (0)
Allocation concealment (selection bias)	24	15 (62.5)	9 (37.5)	0 (0)
Blinding of participants and personnel (performance bias)	24	12 (50)	0 (0)	12 (50)
Blinding of outcome assessment (detection bias)	41	28 (68.3)	6 (14.6)	7 (17.1)
Incomplete outcome data (attrition bias)	24	17 (70.8)	4 (16.7)	3 (12.5)
Other bias	24	17 (70.8)	2 (8.3)	5 (20.8)

a RoB 1: Cochrane risk-of-bias tool version 1.

Among 161 assessments, the first-pass produced invalid outputs in 3(1.9%) and 26 (16.1%) of Gemma 2 base assessments for clinicians 1 and 2, respectively. For TRoB-1, the corresponding count was 2 (1.2%) for each clinician (Multimedia Appendix 6).

Results of the sensitivity analysis excluding invalid base-model outputs and the trial-level sensitivity analysis are shown in Multimedia Appendix 8. We found that the trial-level sensitivity analysis was consistent with the record-level analyses for both clinicians.

**Table 2.** Performance of TRoB-1 and comparative workflows on the external validation set (N=161).^a^

Model	Accuracy, n (%; 95% CI)	Macro-*F*_1,_ (95% CI)	MCC^b^, (95% CI)	Cohen κ, (95% CI)	Gwet AC1^c^, (95% CI)	Final valid output, n (%)
Clinician 1						
Gemma 2 base (strict)	97 (60.2; 51.6%‐68.2%)	0.518 (0.437‐0.585)	0.324 (0.218‐0.428)	—^d^	—	158 (98.1)
TRoB-1 raw	119 (73.9; 68.5%‐79.2%)	0.603 (0.543‐0.649)	0.461 (0.375‐0.545)	0.460 (0.374‐0.540)	0.656 (0.568‐0.739)	161 (100)
Clinician-verified TRoB-1	132 (82.0; 75.8%‐87.9%)	0.709 (0.642‐0.768)	0.615 (0.516‐0.718)	0.614 (0.512‐0.715)	0.765 (0.672‐0.849)	—
Clinician 2						
Gemma 2 base (strict)	84 (52.2; 43.7%‐61.1%)	0.497 (0.414‐0.570)	0.286 (0.190‐0.387)	—	—	136 (84.5)
TRoB-1 raw	118 (73.3; 66.9%‐79.7%)	0.606 (0.531‐0.670)	0.466 (0.371‐0.563)	0.465 (0.369‐0.558)	0.644 (0.544‐0.741)	161 (100)
Clinician-verified TRoB-1	136 (84.5; 79.4%‐89.5%)	0.776 (0.698‐0.834)	0.688 (0.595‐0.775)	0.687 (0.592‐0.771)	0.794 (0.716‐0.867)	—

a Accuracy and valid outputs are reported as n (%). Data are estimates with 95% CIs unless otherwise indicated. For the primary base model, invalid or incomplete outputs were counted as incorrect. Therefore, Cohen κ and Gwet AC1 were not calculated. For macro-*F*_1_, MCC, Cohen κ, and Gwet AC1, the values preceding the 95% CIs are the respective point estimates.

b MCC: Matthews correlation coefficient.

c Gwet AC1: Gwet first-order agreement coefficient.

d Not applicable.

**Figure 3.**
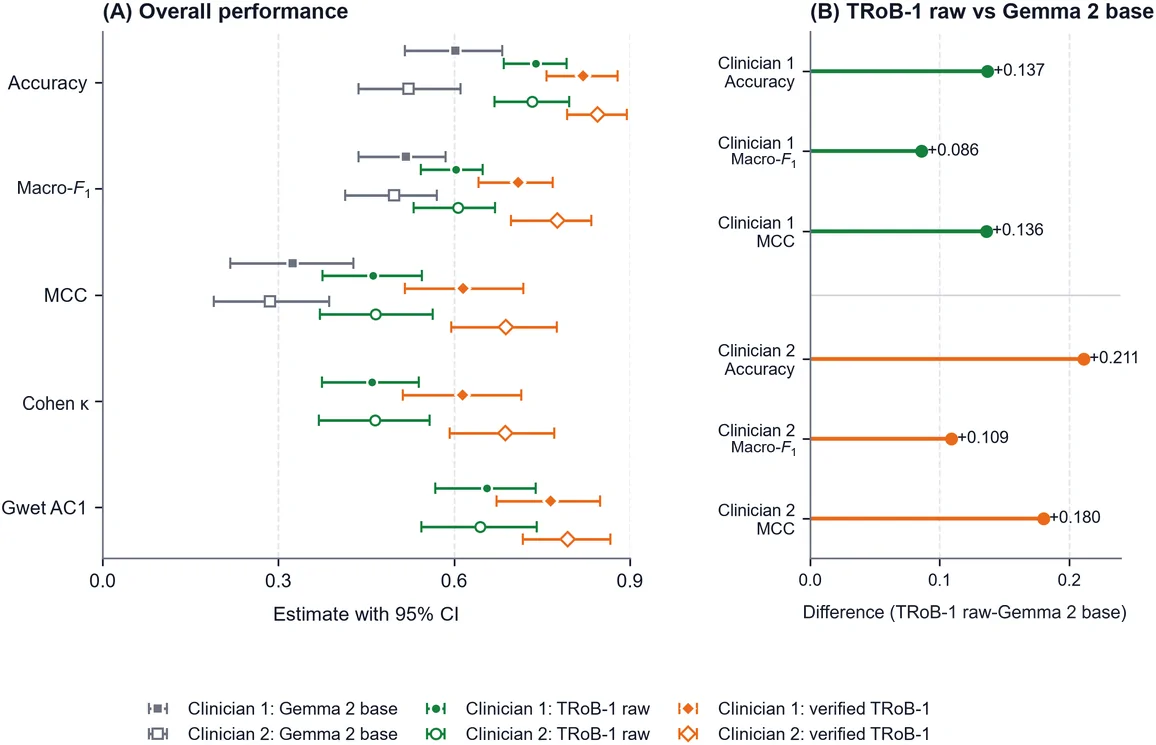
Overall external-validation performance and comparison between TRoB-1 raw outputs and the Gemma 2 base workflow. Panel A presents performance metrics for the Gemma 2 base workflow, TRoB-1 raw outputs, and clinician-verified TRoB-1 outputs. Markers (squares, circles, and diamonds) represent the Gemma 2 base workflow, TRoB-1 raw outputs, and clinician-verified outputs, with horizontal lines indicating corresponding 95% CIs. Panel B shows the differences in accuracy, macro-*F*_1_, and MCC between TRoB-1 raw outputs and the Gemma 2 base workflow. Gwet AC1: Gwet first-order agreement coefficient; MCC: Matthews correlation coefficient.

### Performance Improvement After Verification

Following verification by clinician 1, TRoB-1 outputs matched the reference standard for 132 out of 161 records, raising record-level agreement from 73.9% (n=119) to 82.0% (n=132). Meanwhile, macro-*F*_1_ increased from 0.603 to 0.709, and the MCC rose from 0.461 to 0.615. Agreement with the reference standard also improved, with Cohen κ rising from 0.460 to 0.614 and Gwet AC1 from 0.656 to 0.765. The increase in record-level agreement with the reference standard was statistically significant (8.1%, 95% CI 4.4%‐11.9%; *P*<.001).

Verification by clinician 2 produced a similar pattern but with slightly larger gains. TRoB-1 outputs matched the reference standard for 136 of 161 records, increasing record-level agreement from 73.3% (n=118) to 84.5% (n=136). The macro-*F*_1_-score improved from 0.606 to 0.776, and the MCC increased from 0.466 to 0.688. Cohen κ rose from 0.465 to 0.687, and Gwet AC1 increased from 0.644 to 0.794. The improvement in record-level agreement was also statistically significant (11.2%, 95% CI 6.9%‐15.4%; *P*<.001).

In terms of error correction, verification reduced misclassifications for both clinicians. Of the 27 high RoB judgments defined by the reference standard, mislabels of unclear risk declined from 11 to 7 with clinician 1 and from 9 to 4 with clinician 2, and mislabels of low risk decreased from 1 to 0 and from 2 to 0, respectively (Figure 4).

**Figure 4.**
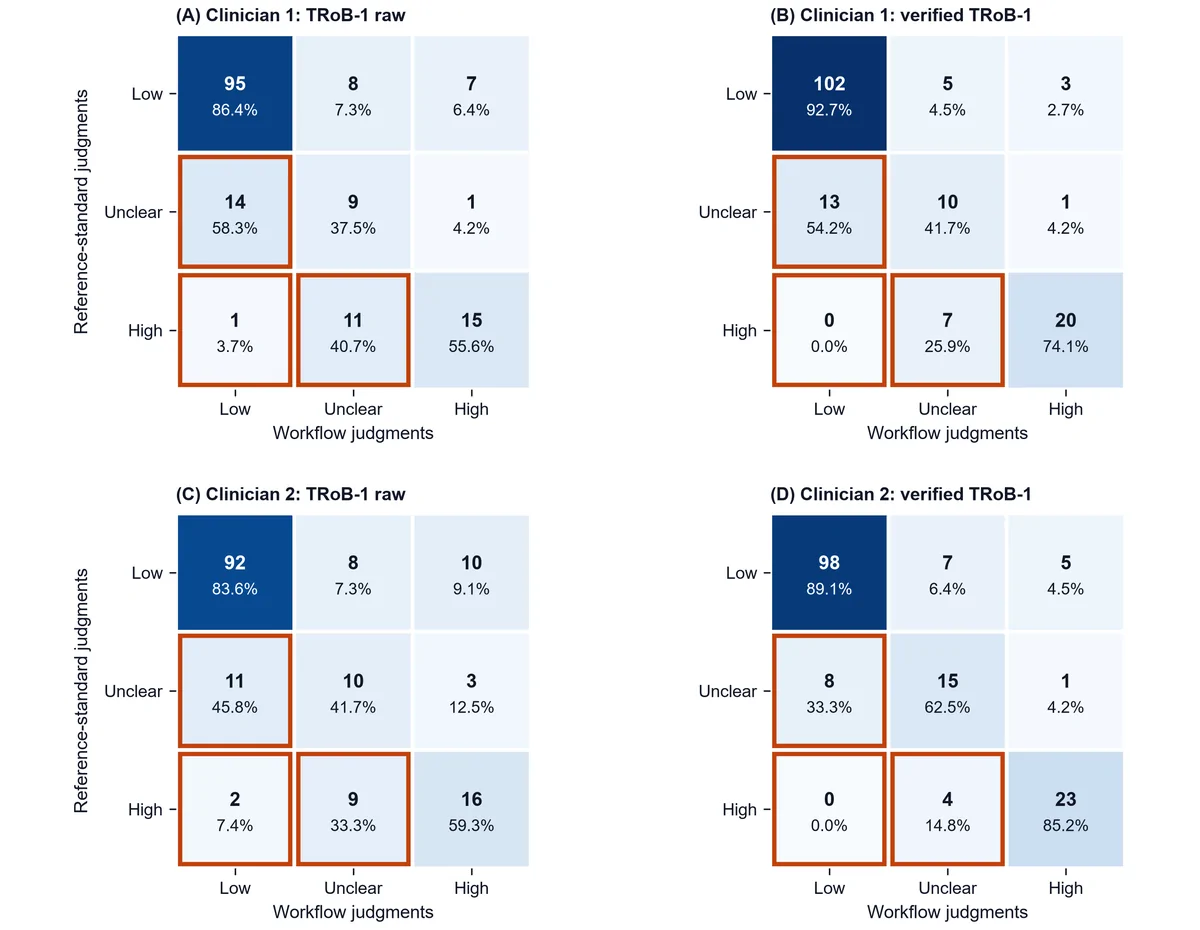
Bias judgments before and after verification. Panels A and C show the row-normalized confusion matrices derived from TRoB-1 raw outputs compared against the human reference standard. Panels B and D display the corresponding matrix for clinician-verified TRoB-1 outputs, with rows representing reference-standard judgments and columns indicating workflow classifications. Values are row-normalized percentages to show class-specific recall. Cells with outlines indicate judgments of unclear-risk or high RoB misclassified as low risk.

### Case-Level Error Correction and Subgroup Performance

Detailed distributions and a deidentified case-level summary are provided in Multimedia Appendix 9.

Class-specific *F*_1_-scores increased after verification. For clinician 1, the *F*_1_-score (95% CI) rose from 0.864 (0.814‐0.907) to 0.907 (0.865‐0.944) for low-risk judgments, from 0.346 (0.188‐0.458) to 0.435 (0.276‐0.557) for unclear-risk judgments, and from 0.600 (0.450‐0.719) to 0.784 (0.651‐0.893) for high-risk judgments (Figure 5). For clinician 2, the corresponding values increased from 0.856 (0.800‐0.903) to 0.907 (0.864‐0.943), from 0.392 (0.189‐0.554) to 0.600 (0.364‐0.759), and from 0.571 (0.400‐0.697) to 0.821 (0.698‐0.915), respectively.

TRoB-1 accuracy before and after clinician verification across clinical areas is shown in Multimedia Appendix 10. The appendix also presents RoB 1 domain-specific accuracy and agreement between the 2 clinicians’ final judgments. The overall agreement between the 2 clinicians was 77.0% (124/161). This estimate reflects agreement across the full clinician-assisted workflow rather than verification alone. Passage selection differed between clinicians and also affected the model outputs.

**Figure 5.**
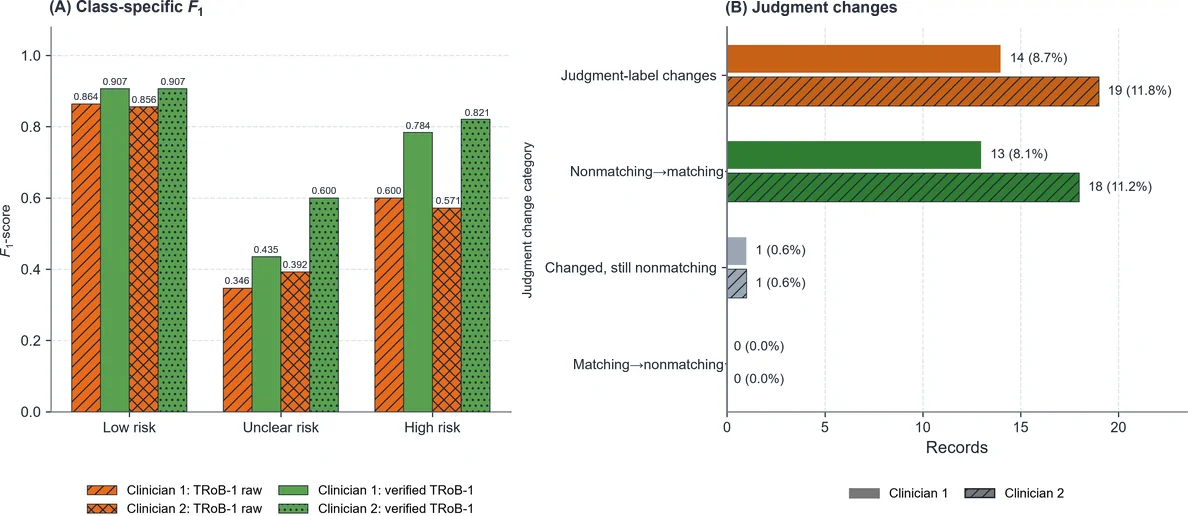
Class-specific performance and modifications. Panel A compares class-specific *F*_1_-scores between TRoB-1 raw outputs and clinician-verified TRoB-1 outputs. Panel B summarizes the modification of judgment during verification for each clinician as well as effects on agreement with the reference standard.

### Time Efficiency of TRoB-1 Workflow

Two independent reviewers spent 947 and 1088 minutes, respectively, on the initial assessment across 24 RCTs, with average durations of 39.5 and 45.3 minutes per RCT. In the TRoB-1 workflow, clinician 1 used 330.0 minutes for supporting passage identification and an additional 52.3 minutes for model output verification for the same set of RCTs. Meanwhile, the automated inference process required 72.9 minutes. The complete TRoB-1 workflow required 455.2 minutes for clinician 1 and 488.4 minutes for clinician 2 (Figure 6).

**Figure 6.**
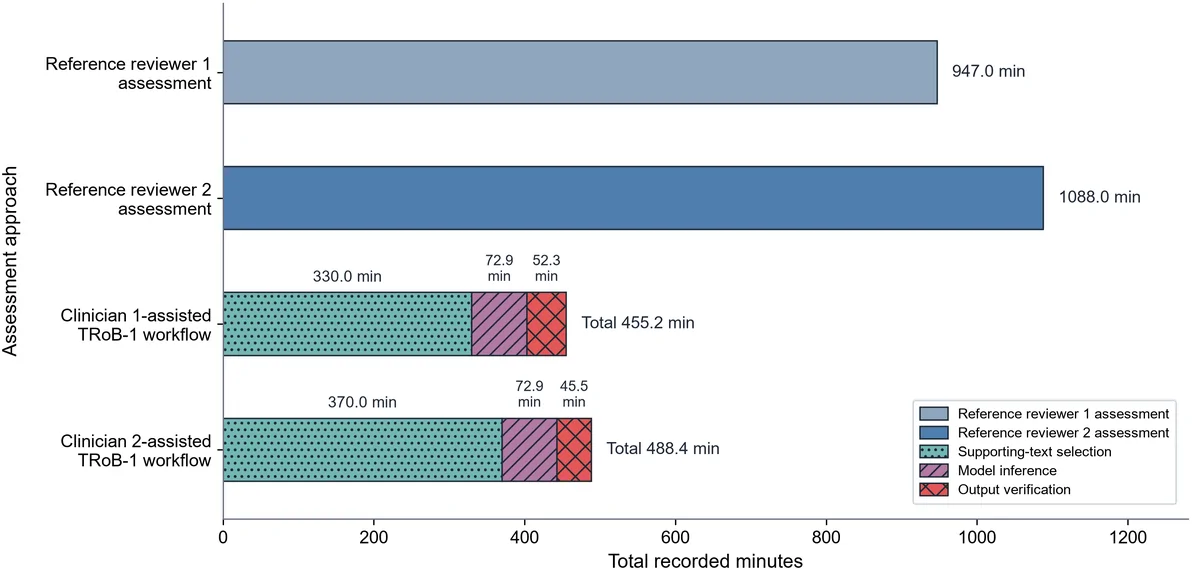
Time requirements for risk of bias (RoB) assessment by human reviewers and the TRoB-1 workflow. Total TRoB-1 workflow time comprises source-passage selection, model inference, and reviewer verification.

### TRoB-1 Web Interface

The overview of the TRoB-1 web interface displayed a summary of the 161 external validation records, including information on completed assessments, records assigned high priority for reviewer verification, average inference time per record, and the distribution of preliminary judgments (Figure 7).

Screenshots of the single-trial assessment, batch assessment, review queue, and analytics page, as well as verification and export functions, are provided in Multimedia Appendix 11.

**Figure 7.**
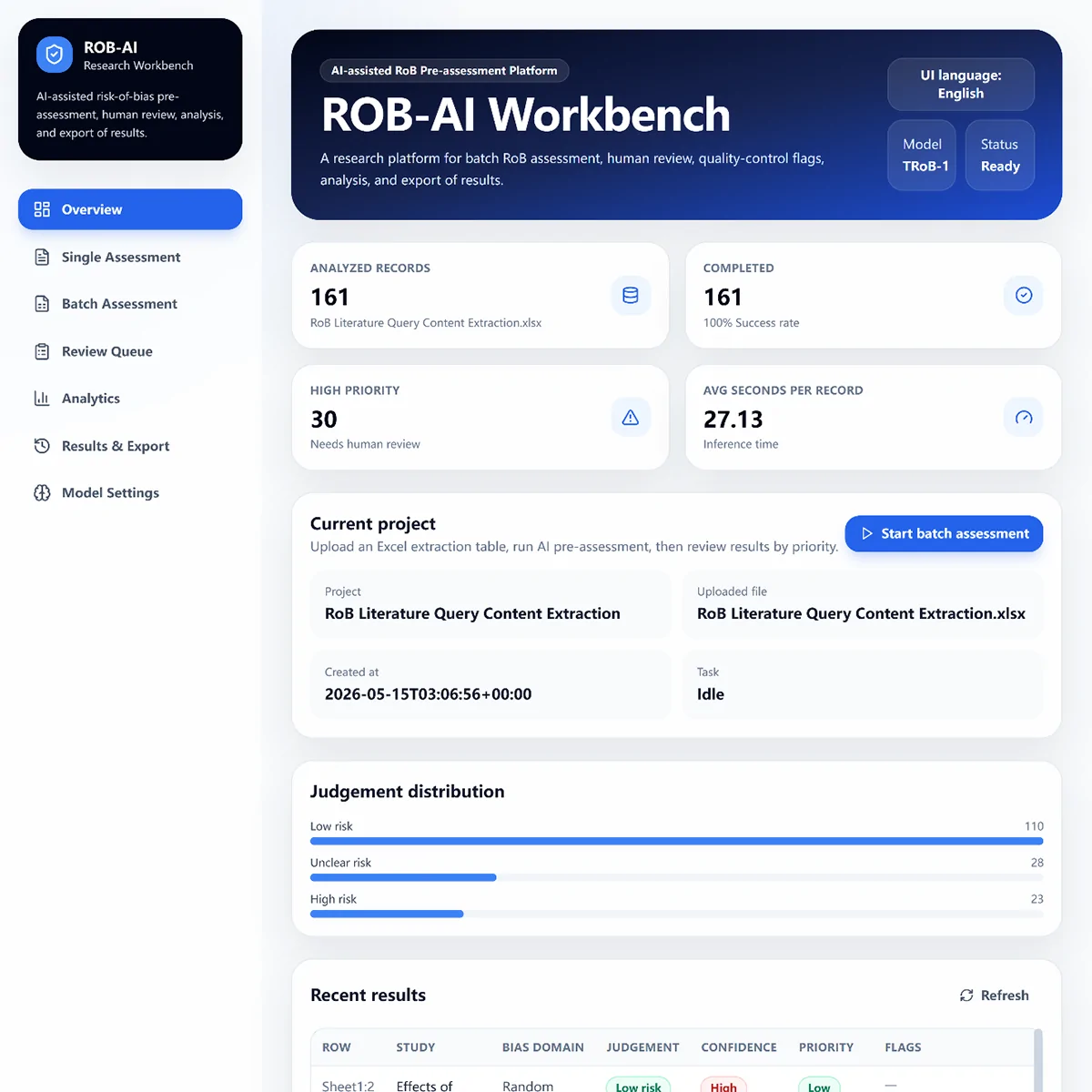
Overview page of the TRoB-1 web interface used for external validation.

### Reproducibility Across Hardware Environments and Repeated Processing

Of 322 inputs, the original server processing produced valid outputs for all 322 (100%) inputs, whereas laptop processing produced 321 (99.7%). At the individual-record level, the 2 environments produced the same RoB 1 judgment for 319 (99.1%) inputs and identical supporting quotations for 314 (97.5%). Only 3 judgment labels differed. A repeat of step 1 on the same laptop reproduced all 322 responses exactly. Hardware and software environments, as well as detailed results, are shown in Multimedia Appendix 12.

## Discussion

### Principal Findings

This study developed and externally validated TRoB-1, a reviewer-supported tool designed to facilitate RoB 1 assessment. Our principal findings demonstrate that TRoB-1 could achieve both efficiency and methodological rigor, offering a practical solution for large-scale and dynamically updating evidence synthesis.

First, we found that domain-specific adaptation is critical for performance improvement. The task-specific fine-tuned TRoB-1 model demonstrated better overall performance than its base Gemma 2 counterpart, with higher accuracy, macro-*F*_1_, and MCC. Living systematic reviews and broad-scope evidence syntheses require frequent updates or the processing of a large amount of literature, which imposes heavy strain on researchers [24]. Given its ability to deliver more accurate preliminary judgments, the model reduces the time and cognitive load required for them.

Second, the integrated reviewer verification module proved to be an essential component of the workflow’s reliability. Our analysis revealed that reviewer verification corrected a substantial proportion of initial misclassifications without introducing new errors. The approach ensures that the appraisal process benefits from both the model’s rapid inference and the reviewers’ expertise, thus enhancing the efficiency and credibility of the assessment. This experience is consistent with broader evidence from clinical LLM applications, where even high-performing models have been found to require human verification to prevent errors [25].

Third, performance analysis by judgment category identified potential difficulties during RoB assessment, particularly with “unclear-risk” classifications. While the model performed better on “low-risk” and “high-risk” judgments, its poor performance on “unclear-risk” reflects an inherent issue within the evidence itself. This challenge often arises from trial reports lacking adequate details, such as incomplete descriptions of key methodological features [26]. Incomplete reporting may account for the lower *F*_1_-scores for unclear-risk judgments after clinician verification. In addition to informing model development, our findings hold important implications for trial investigators. After verification, some reference-unclear records were rated low risk by both clinicians. Rating unclear records as low risk may make the evidence appear of higher quality than it is. However, because no high-risk record was changed to low risk, these misclassifications are unlikely to change conclusions that mainly depend on high-risk vs low-risk judgments. Therefore, trial investigators should be encouraged not only to register the trials, but also to ensure that methodological details and data are publicly accessible.

Fourth, TRoB-1 returned a supporting quotation with each preliminary judgment. To generate the quotation, reviewers first selected a relevant passage from the trial report. TRoB-1 then identified a specific quotation within that passage to support its judgment. This workflow reduced the manual effort required for sentence-level evidence identification and facilitated reviewer verification. Studies have also shown that collaborative LLM workflows can achieve accuracy comparable to manual extraction [27,28]. By linking each preliminary judgment to a specific quotation, the workflow provided a transparent and traceable basis for evidence review, rather than only a structured classification output. Reviewers could review, retain, or revise both the judgment and the supporting quotation, and all modifications were recorded.

### Comparison With Prior Studies

A recent scoping review of automation methods for systematic reviews found that only 7.3% of identified research focused on RoB assessment, while the majority concentrated on literature screening [29]. Despite the development of automation technology, real-world adoption remains limited by usability barriers, maintenance demands, and incomplete reporting of their technical specifications [29,30]. The transfer of technological progress into practice remains particularly weak for complex tasks such as RoB assessment, which requires both critical reasoning and standardized classification. TRoB-1 bridges the gap by a validated, semiautomated workflow integrating efficient domain-specific fine-tuning and reliable reviewer verification.

Although early automation attempts showed that machine learning systems could assist with locating supporting text and generating RoB predictions, their performance varied across bias domains and review settings [9]. Subsequently, a growing number of studies have turned to general-purpose LLMs. In a study using ChatGPT-4o (OpenAI) with prompt engineering, the model achieved only 50.7% agreement with reviewers for overall RoB assessment [31]. Similarly, Claude 3.5 Sonnet (Anthropic), using structured prompting for RoB assessment without task-specific training, showed feasibility but also revealed the limitations of prompt-based methods, which depend entirely on the model’s preexisting knowledge and instruction quality [32]. Another study further concluded that off-the-shelf LLMs, prompted with or without annotated examples, are not yet effective for automating RoB assessments [33]. These findings indicate the shortcomings of prompt-only strategies for tackling complex analytical tasks. By comparison, our TRoB-1 workflow adopts supervised fine-tuning with a rigorously constructed training set, which effectively mitigates these drawbacks.

Researchers have recently begun to use transformer-based models for domain-specific adaptation. Risk of Bias Inference, for instance, was developed to automate RoB assessment, reporting an area under the receiver operating characteristic curve of 0.83 [34]. However, it was trained on a small, automatically annotated dataset with limited human validation and notable class imbalance. While automation has progressed for evidence synthesis, most existing systems focus on data extraction rather than RoB judgment [35]. In particular, RoB assessment studies either rely on general LLMs or treat RoB as a secondary outcome following data extraction [36,37]. In contrast to prior work, TRoB-1 is explicitly adapted to perform RoB assessment for the Cochrane RoB 1 tool. This adaptation allowed us to distinguish raw model outputs from reviewer-verified judgments and quantify the contributions of fine-tuning and human verification. Accordingly, we adopted macro-*F*_1_ and class-specific measures to prevent high overall accuracy from obscuring poor performance in minority categories [38].

Evidence from other clinical scenarios supports our choice of task-specific adaptation. Trained models for domain-specific tasks have been shown to be more effective than general-purpose LLMs for targeted tasks [39]. In a clinical classification setting, an untuned Llama 3 (Meta) performed almost no better than random chance, whereas low-rank adaptation fine-tuning substantially enhanced performance [40]. The fine-tuned model could also generate explanations that strengthened decision transparency. Likewise, a study showed that general-purpose LLMs lacked the specialized knowledge required for reliable clinical decisions, and domain adaptation could improve performance [41].

### Implications for Practice

For researchers of systematic reviews and evidence synthesis, TRoB-1 makes RoB assessment more efficient and effective. TRoB-1 does not retrieve evidence; instead, it extracts source text from reviewer-selected passages to generate preliminary judgments and supporting quotations. Therefore, researchers can spend less time searching for supporting quotations and better applying their expertise to final judgment. Furthermore, since TRoB-1 provides quotations for each judgment, researchers can easily check the evidence basis for RoB assessment. This is especially critical for systematic reviews that inform clinical guideline development or health care decision-making, where every judgment must be transparent and justifiable.

TRoB-1 is particularly valuable in living systematic reviews, as new RCTs become available regularly and RoB assessment must be performed on an ongoing basis. The tool accelerates the assessment of each new study, thus making it easier for researchers to keep reviews up-to-date. By providing a uniform workflow for each assessment, the tool supports consistent documentation of RoB judgments and revisions across review updates.

Beyond its utility for RoB assessment, the TRoB-1 workflow offers a practical framework for broader integration of task-specific LLMs into evidence synthesis. Its hybrid workflow combining domain-specific fine-tuning and reviewer verification can be adapted to other types of evidence review tasks. This collaborative approach also serves as a replicable model for developing trustworthy LLM tools tailored to clinical practice.

### Strengths

The present study has several strengths worth highlighting. First, TRoB-1 was developed to address shortcomings in recent studies that attempted to use LLMs to facilitate RoB assessment, most of which relied on prompt engineering instead of task-specific fine-tuning. Our study addressed this gap by using domain-specific fine-tuning across all bias domains. Second, few studies incorporate human verification beyond a post hoc check. However, human expert review is embedded throughout the entire workflow development and inference process. All annotated records used for model training underwent rigorous reviewer validation during dataset construction. Furthermore, the human verification module is retained in the final TRoB-1 workflow. This step successfully corrects mismatched judgments without introducing new errors, balancing efficiency with robust reliability.

Third, the external validation set was strictly separated from model development. It included RCTs in a broader range of clinical areas, enabling an exploratory analysis of generalizability. Fourth, the three-arm comparison, involving the base Gemma 2 model, the raw fine-tuned outputs, and the clinician-verified judgments, allowed us to distinguish the impact of algorithmic fine-tuning from expert human verification. Finally, we implemented TRoB-1 in a locally hosted, browser-based interface that brought model-assisted assessment and reviewer verification into a single workflow. The interface allowed reviewers to easily use the system without directly interacting with the complex model code.

### Limitations

Despite the strengths, several limitations should be acknowledged. First, the external validation set was based on 24 RCTs involving 161 assessment records. While this sample size is comparable to that of previous studies, further application of TRoB-1 is deserved in systematic reviews and routine practice. Second, the study focuses on RoB 1, and findings may not directly generalize to other RoB assessment settings. Future studies should therefore explore the applicability of the TRoB-1 workflow to other quality appraisal tools. Third, deploying TRoB-1 requires access to computational resources for fine-tuning and inference, which may not be readily available to all systematic review researchers. Future studies should optimize the model to reduce computational demands, enabling deployment on standard hardware while maintaining performance. Fourth, both clinicians viewed Step 2 explanations that were generated to support the model’s preliminary judgments. This may have influenced clinicians’ final judgments during verification. Future studies could compare AI-assisted and blinded human-only assessments. In addition, the external validation sample was exploratory because of the limited number of included RCTs. Future studies with larger and more diverse validation samples are needed. Finally, TRoB-1 only assessed reviewer-selected passages. Information missing from these passages might be reported elsewhere in the trial report. This limitation may partly explain the persistently lower *F*_1_-scores for unclear-risk judgments after verification.

### Conclusions

By integrating structured judgments, supporting quotations, and clinician verification, TRoB-1 provides a feasible reviewer-support workflow for RoB 1 assessment. The results support the use of task-specific fine-tuning and clinician verification to produce traceable judgments. Unlike prior approaches that primarily rely on prompt engineering, TRoB-1 offers a practical solution for regular RoB assessment in living systematic reviews and large-scale evidence synthesis. By linking each judgment to a supporting quotation, the workflow ensures that every assessment is traceable and verifiable. The TRoB-1 web interface makes this functionality readily accessible, providing a user-friendly platform where reviewers can view, revise, and export assessments without requiring specialized technical skills.

## Supplementary material

10.2196/108001 Multimedia Appendix 1 Training environment and fine-tuning settings.

10.2196/108001 Multimedia Appendix 2 Literature search for the construction of external validation sets.

10.2196/108001 Multimedia Appendix 3 Rule-based generation and distribution of candidate evidence hints.

10.2196/108001 Multimedia Appendix 4 Domain-specific RoB (risk of bias) assessment rules.

10.2196/108001 Multimedia Appendix 5 Two-step prompt templates and expected outputs.

10.2196/108001 Multimedia Appendix 6 Inference settings and output checks.

10.2196/108001 Multimedia Appendix 7 Statistical analysis software and package versions.

10.2196/108001 Multimedia Appendix 8 Performance comparisons and sensitivity analyses.

10.2196/108001 Multimedia Appendix 9 Case-level analysis of judgment discrepancies in the 2 clinician-assisted runs.

10.2196/108001 Multimedia Appendix 10 Stratified performance and between-clinician agreement.

10.2196/108001 Multimedia Appendix 11 Additional views of the TRoB-1 web interface.

10.2196/108001 Multimedia Appendix 12 Reproducibility across runs and hardware environments revised.

10.2196/108001 Checklist 1 TRIPOD-LLM checklist.

## Abbreviations

COPD: chronic obstructive pulmonary disease
Gwet AC1: Gwet first-order agreement coefficient
LLM: large language model
MCC: Matthews correlation coefficient
QLoRA: quantized low-rank adaptation
RCT: randomized controlled trial
RoB: risk of bias
RoB 1: Cochrane risk-of-bias tool version 1
TRIPOD-LLM: Transparent Reporting of a Multivariable Model for Individual Prognosis or Diagnosis–Large Language Model

## References

[1] Fanaroff AC, Fudim M, Califf RM, Windecker S, Smith SC, Lopes RD (2020). Levels of evidence supporting drug, device, and other recommendations in the American Heart Association/American College of Cardiology guidelines. Am Heart J.

[2] Cazzola M, Stolz D, Bafadhel M, Rogliani P (2024). Understanding evidence from randomised controlled trials and meta-analyses: a comparative overview. Eur Respir J.

[3] Higgins JPT, Altman DG, Gøtzsche PC (2011). The Cochrane Collaboration's tool for assessing risk of bias in randomized trials. BMJ.

[4] Minozzi S, Cinquini M, Gianola S, Gonzalez-Lorenzo M, Banzi R (2020). The revised Cochrane risk of bias tool for randomized trials (RoB 2) showed low interrater reliability and challenges in its application. J Clin Epidemiol.

[5] Crocker TF, Lam N, Jordão M (2023). Risk-of-bias assessment using Cochrane’s revised tool for randomized trials (RoB 2) was useful but challenging and resource-intensive: observations from a systematic review. J Clin Epidemiol.

[6] Moore THM, Higgins JPT, Dwan K (2023). Ten tips for successful assessment of risk of bias in randomized trials using the RoB 2 tool: early lessons from Cochrane. Cochrane Evid Synth Methods.

[7] Babić A, Barcot O, Visković T (2024). Frequency of use and adequacy of Cochrane risk of bias tool 2 in non-Cochrane systematic reviews published in 2020: meta-research study. Res Synth Methods.

[8] Marshall IJ, Kuiper J, Wallace BC (2016). RobotReviewer: evaluation of a system for automatically assessing bias in clinical trials. J Am Med Inform Assoc.

[9] Tian Y, Yang X, Doi SA (2024). Towards the automatic risk of bias assessment on randomized controlled trials: a comparison of RobotReviewer and humans. Res Synth Methods.

[10] Jardim PSJ, Rose CJ, Ames HM, Echavez JFM, Van de Velde S, Muller AE (2022). Automating risk of bias assessment in systematic reviews: a real-time mixed methods comparison of human researchers to a machine learning system. BMC Med Res Methodol.

[11] Soboczenski F, Trikalinos TA, Kuiper J, Bias RG, Wallace BC, Marshall IJ (2019). Machine learning to help researchers evaluate biases in clinical trials: a prospective, randomized user study. BMC Med Inform Decis Mak.

[12] Arno A, Thomas J, Wallace B, Marshall IJ, McKenzie JE, Elliott JH (2022). Accuracy and efficiency of machine learning–assisted risk-of-bias assessments in “real-world” systematic reviews: a noninferiority randomized controlled trial. Ann Intern Med.

[13] Qureshi R, Shaughnessy D, Gill KAR, Robinson KA, Li T, Agai E (2023). Are ChatGPT and large language models “the answer” to bringing us closer to systematic review automation?. Syst Rev.

[14] Bedi S, Liu Y, Orr-Ewing L (2025). Testing and evaluation of health care applications of large language models: a systematic review. JAMA.

[15] Hu EJ, Shen Y, Wallis P, Allen-Zhu Z, Li Y, Wang S LoRA: low-rank adaptation of large language models. https://openreview.net/challenge?redirect=%2Fpdf%3Fid%3DnZeVKeeFYf9.

[16] Dettmers T, Pagnoni A, Holtzman A, Zettlemoyer L QLoRA: efficient finetuning of quantized LLMs.

[17] Li Y, Yang F, Wu M, Li J (2026). Human-AI collaboration enhances the performance of large language models in risk of bias assessment. BMC Med Res Methodol.

[18] Gallifant J, Afshar M, Ameen S (2025). The TRIPOD-LLM reporting guideline for studies using large language models. Nat Med.

[19] Saric F, Barcot O, Puljak L (2019). Risk of bias assessments for selective reporting were inadequate in the majority of Cochrane reviews. J Clin Epidemiol.

[20] Higgins JPT, Altman DG, Higgins JPT, Green S (2008). Cochrane Handbook for Systematic Reviews of Interventions.

[21] Gorodkin J (2004). Comparing two K-category assignments by a K-category correlation coefficient. Comput Biol Chem.

[22] Gwet KL (2008). Computing inter-rater reliability and its variance in the presence of high agreement. Br J Math Stat Psychol.

[23] Vach W, Gerke O (2023). Gwet’s AC1 is not a substitute for Cohen’s kappa - a comparison of basic properties. MethodsX.

[24] Nevill CR, Sharifan A, O’Mahony A (2025). Capturing the influx of living systematic reviews: a systematic methodological survey. J Clin Epidemiol.

[25] Silveira Nogueira Reis Z, Tuler Albergaria E, Silvina Pagano A (2026). Large language model-generated patient instructions for prescriptions in primary health care: preclinical algorithm validation. J Med Internet Res.

[26] Hróbjartsson A, Boutron I, Turner L, Altman DG, Moher D (2013). Assessing risk of bias in randomized clinical trials included in Cochrane reviews: the why is easy, the how is a challenge. Cochrane Database Syst Rev.

[27] Kasireddy E, Chow C, Collet J, Pourrahmat MM, Fazeli MS (2026). Evaluating the performance of Claude 3.7 Sonnet in data extraction automation for systematic literature reviews. Value Health Reg Issues.

[28] Li Y, Datta S, Rastegar-Mojarad M (2025). Enhancing systematic literature reviews with generative artificial intelligence: development, applications, and performance evaluation. J Am Med Inform Assoc.

[29] Tóth B, Berek L, Gulácsi L, Péntek M, Zrubka Z (2024). Automation of systematic reviews of biomedical literature: a scoping review of studies indexed in PubMed. Syst Rev.

[30] Marshall IJ, Wallace BC (2019). Toward systematic review automation: a practical guide to using machine learning tools in research synthesis. Syst Rev.

[31] Taneri PE (2025). Human versus artificial intelligence: comparing Cochrane authors’ and ChatGPT’s risk of bias assessments. Cochrane Evid Synth Methods.

[32] Huang J, Lai H, Zhao W (2025). Large language model–assisted risk-of-bias assessment in randomized controlled trials using the revised risk-of-bias tool: evaluation study. J Med Internet Res.

[33] Šuster S, Baldwin T, Verspoor K (2024). Zero- and few-shot prompting of generative large language models provides weak assessment of risk of bias in clinical trials. Res Synth Methods.

[34] Dias AC, Moreira VP, Comba JLD (2025). RoBIn: a transformer-based model for risk-of-bias inference with machine reading comprehension. J Biomed Inform.

[35] Gartlehner G, Kahwati L, Hilscher R (2024). Data extraction for evidence synthesis using a large language model: a proof-of-concept study. Res Synth Methods.

[36] Barsby J, Hume S, Lemmey HA, Cutteridge J, Lee R, Bera KD (2025). Pilot study on large language models for risk-of-bias assessments in systematic reviews: A(I) new type of bias?. BMJ Evid Based Med.

[37] Lai H, Liu J, Bai C (2025). Language models for data extraction and risk of bias assessment in complementary medicine. NPJ Digit Med.

[38] Pan C, Lu W, Chen B, Zhang G, Yang Z, Hao J (2025). Automated literature screening for hepatocellular carcinoma treatment through integration of 3 large language models: methodological study. JMIR Med Inform.

[39] Brown KE, Yan C, Li Z (2025). Large language models are less effective at clinical prediction tasks than locally trained machine learning models. J Am Med Inform Assoc.

[40] Chen MC, Ruan SJ, Wu JH, Chen PF (2026). Classifying American Society of Anesthesiologists physical status with a low-rank-adapted large language model: development and validation study. J Med Internet Res.

[41] Zhang Y, Song J, Bi C (2026). Benchmarking large language models and prompt engineering strategies in microsatellite instability cancers: evaluation study. J Med Internet Res.

[42] lyt9906/TRoB-1. Hugging Face.

