# Supplementary material for: A Fine-Tuned Large Language Model Workflow to Support Risk of Bias Assessment: Development, External Validation, and Reviewer Verification Study
Source: J Med Internet Res. 2026 Nov 13;28:e108001. doi: 10.2196/108001 (PMC13765787; doi:10.2196/108001)

Additional Screenshots of the TRoB-1 Web Interface

A. Single-Trial Assessment

The single-trial assessment module enables researchers to assess the RoB of one randomized controlled trial per session.

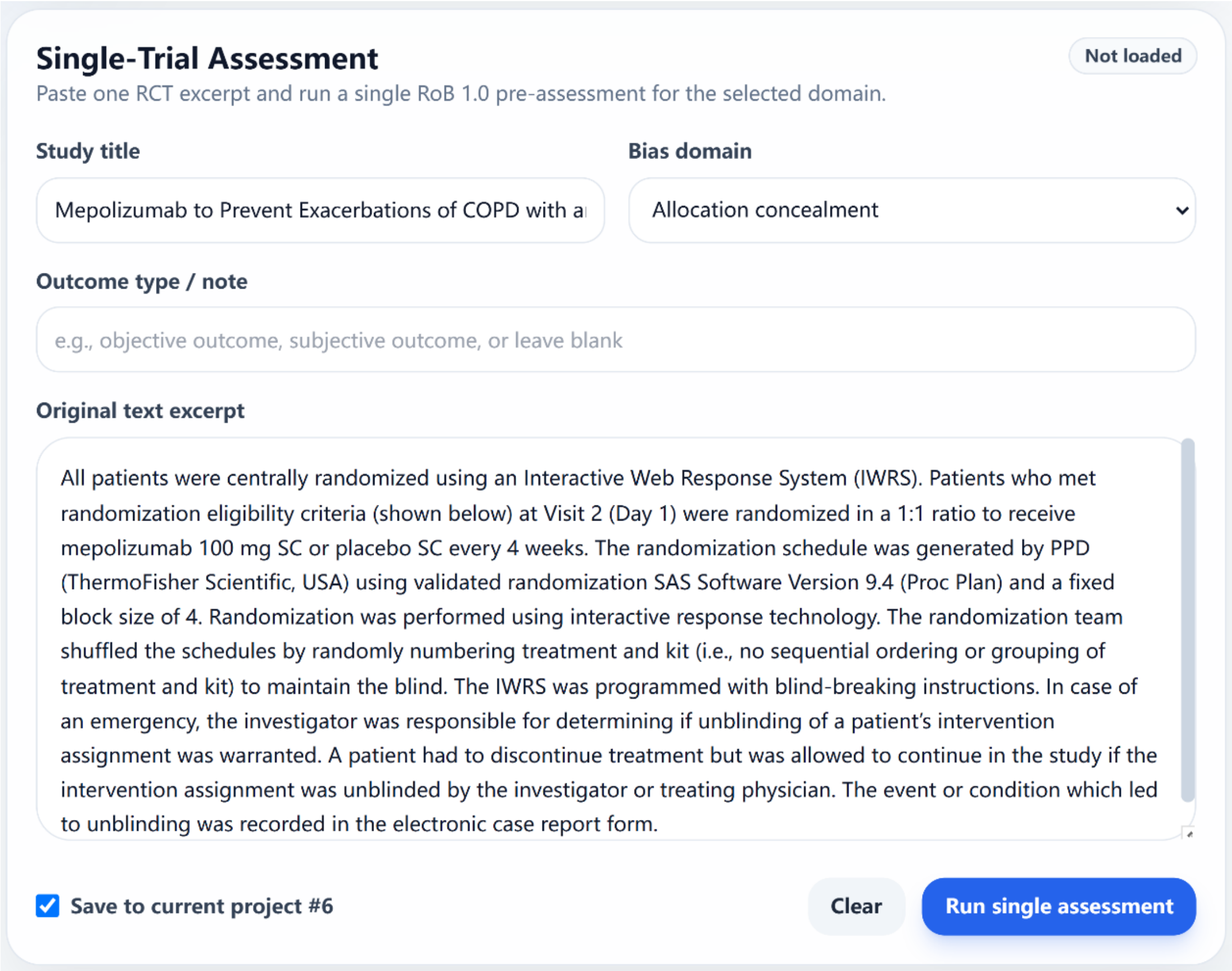

B. Batch assessment

The batch assessment module allows reviewers to upload an XLSX, XLS, or CSV file containing records from multiple trials. As a minimum requirement, the uploaded data must include the target RoB domain and the corresponding source text. After processing is complete, a concise record-level summary will be displayed below the upload area.

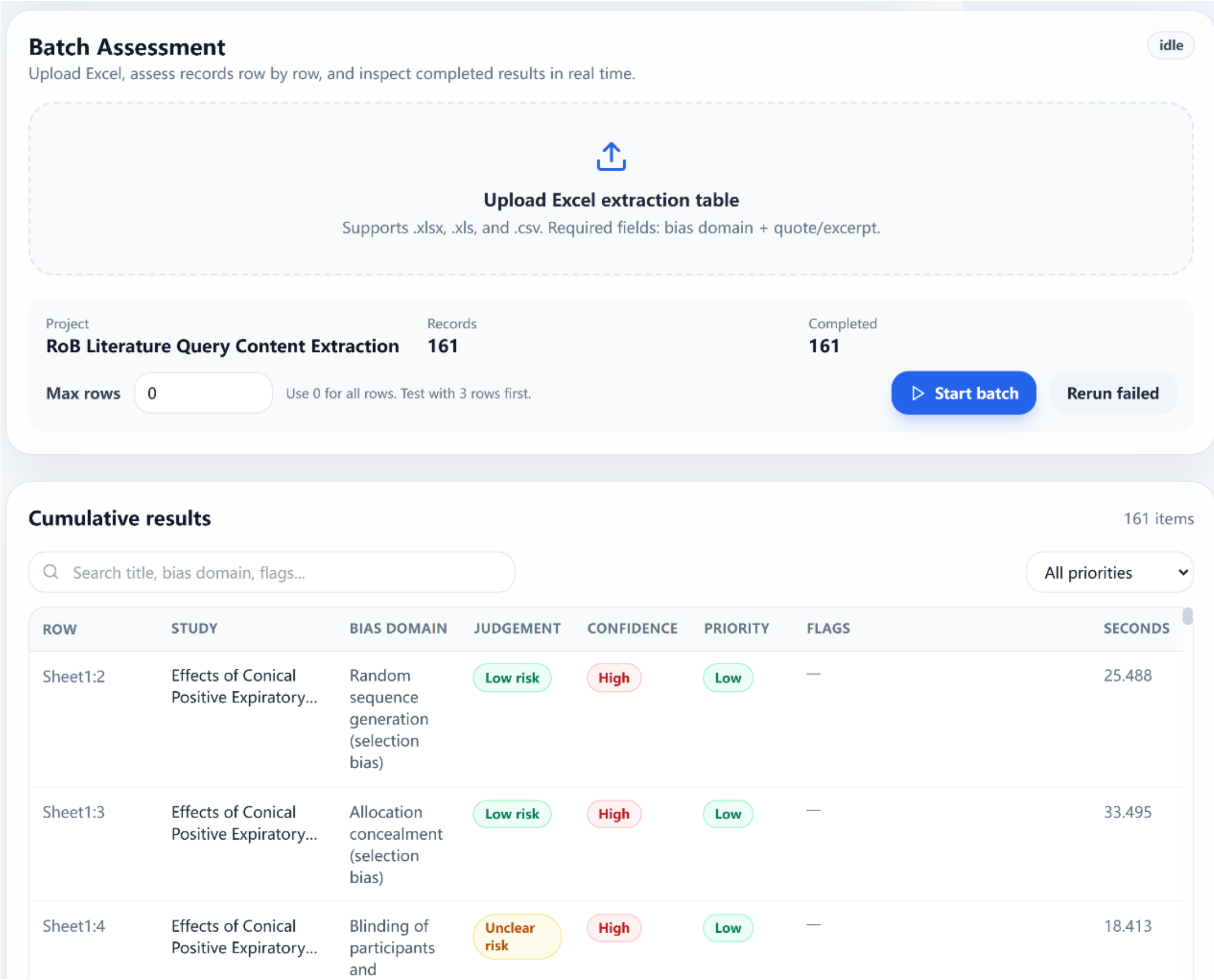

C. Review queue

The review queue presents each preliminary judgement together with the model explanation and supporting quotation. Reviewers can verify the assessment, indicate whether they accept the model judgement, and, when they disagree, revise the judgement and document the reason for the change.

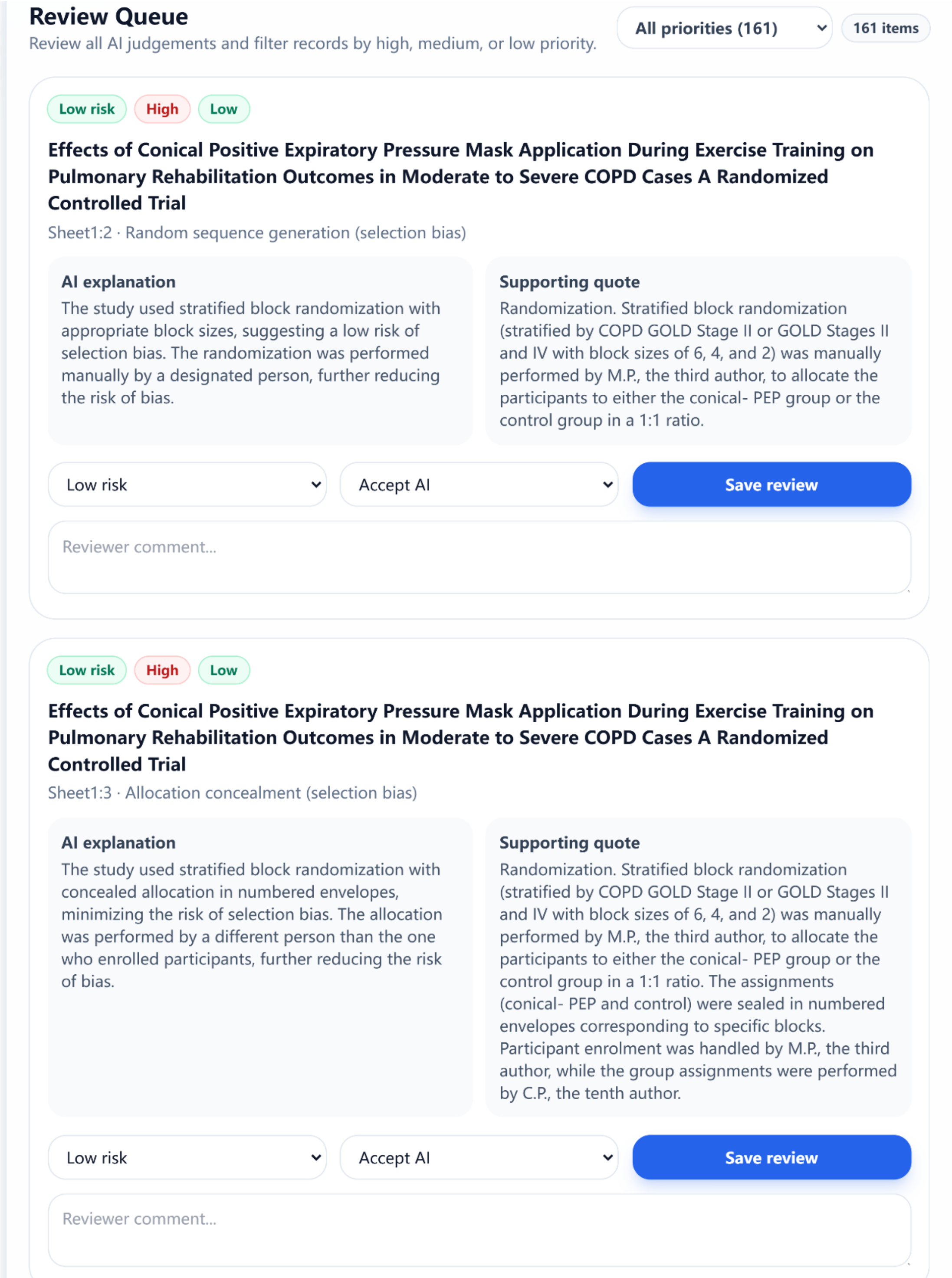

D. Audit and export

The audit and export module provides searchable access to the reviewed records. Reviewer-verified results can be downloaded in XLSX and CSV formats, and the audit log available in JSON Lines (JSONL) format.

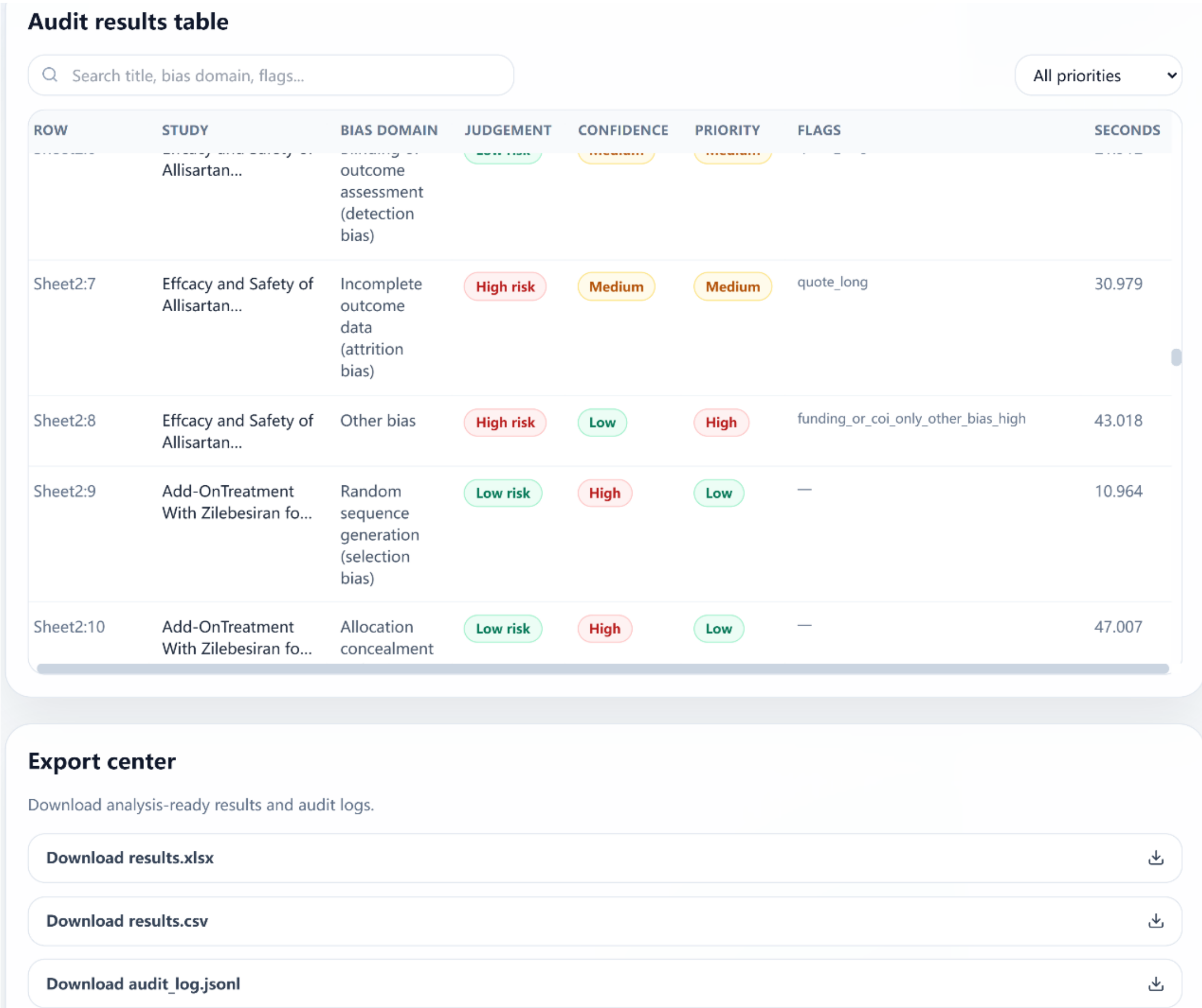

E. Analytics

The analytics module summarizes assessment completion, processing time, the distribution of preliminary judgements, and the number of assessed records in each RoB 1 domain.

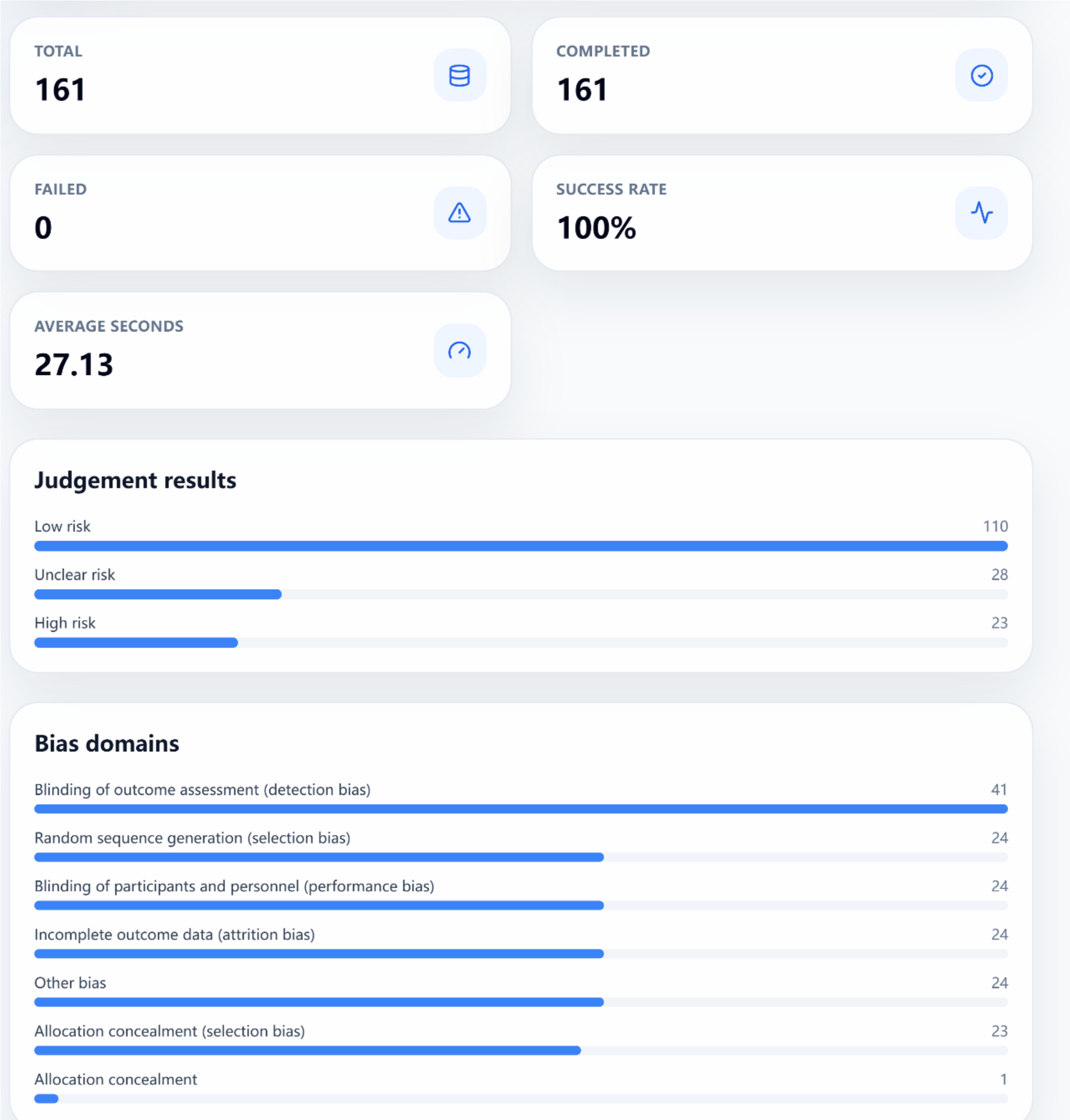

Supplement: Supplementary file 1 [file jmir-v28-e108001-s011.pdf]
