# Supplementary material for: A Fine-Tuned Large Language Model Workflow to Support Risk of Bias Assessment: Development, External Validation, and Reviewer Verification Study
Source: J Med Internet Res. 2026 Nov 13;28:e108001. doi: 10.2196/108001 (PMC13765787; doi:10.2196/108001)
Supplement: Supplementary file 3 [file jmir-v28-e108001-s001.docx]

**Training Environment and Fine-Tuning Settings**

**Table S3. Model training environment**

| **Component** | **Software** | **Version** |
| --- | --- | --- |
| Operating system | Ubuntu | 22.04 |
| Graphics processor | NVIDIA GeForce RTX 4090 | 24 GB memory |
| Training framework | LLaMA-Factory | 0.9.5.dev0 |
| Deep-learning framework | PyTorch | 2.10.0+cu128 |
| CUDA | CUDA | 12.8 |
| Model library | Transformers | 4.57.6 |
| Adapter library | PEFT | 0.18.1 |
| Quantization library | bitsandbytes | 0.49.2 |

**Table S4. Two-stage fine-tuning settings**

| **Parameter** | **Stage 1** | **Stage 2** |
| --- | --- | --- |
| Starting model | Gemma 2-9B-Instruct | Stage 1 adapter |
| Training purpose | RoB 1 judgement | Structured-output adherence |
| Method | 4-bit QLoRA supervised fine-tuning | 4-bit QLoRA continued fine-tuning |
| LoRA rank | 32 | 32 |
| LoRA alpha | 64 | 64 |
| LoRA dropout | 0.05 | 0.05 |
| Target modules | q, k, v, o, up, down, and gate projection layers | Same as stage 1 |
| Learning rate | 1e-4 | 5e-6 |
| Training epochs | 3 | 2 |
| Maximum sequence length | 2048 tokens | 2048 tokens |
| Batching | Batch size 1; gradient accumulation 8 | Batch size 1; gradient accumulation 8 |
| Learning-rate schedule | Cosine | Cosine; warm-up ratio 0.05 |
| Optimizer | AdamW | Paged AdamW, 8-bit |
| Precision | bfloat16 | bfloat16 |
| Random seed | 42 | 42 |
