# Supplementary material for: A Fine-Tuned Large Language Model Workflow to Support Risk of Bias Assessment: Development, External Validation, and Reviewer Verification Study
Source: J Med Internet Res. 2026 Nov 13;28:e108001. doi: 10.2196/108001 (PMC13765787; doi:10.2196/108001)
Supplement: Supplementary file 4 [file jmir-v28-e108001-s002.docx]

Literature Search for the Construction of External Validation Sets

**Inclusion and Exclusion Criteria**

Eligible studies were randomized controlled trials (RCTs) conducted in patients with chronic obstructive pulmonary disease (COPD), diabetes, hypertension, or any of the prespecified out-of-domain clinical areas that were not represented in the training set. The out-of-domain areas included dermatology, rheumatology, oncology, obstetrics/pre-eclampsia, trauma/emergency/neurosurgical, hepatology/metabolic liver disease. Only full-text publications reporting findings of the trials were eligible.

Nonrandomized controlled trials, observational studies, study protocols without full-text publication, secondary or post hoc analyses that did not report the primary trial results, literature reviews, conference abstracts, and duplicate publication. When multiple literature described the same RCT, the primary report was used for validation.

**Table S5. Search Summary**

| **Clinical areas** | **Search terms for diseases** | **Records retrieved** |
| --- | --- | --- |
| COPD | COPD, chronic obstructive pulmonary disease | 226 |
| Diabetes | Diabetes mellitus, diabetes-related, glycemic, hyperglycemia | 1745 |
| Hypertension | Hypertension, high blood pressure, elevated blood pressure, arterial hypertension | 648 |
| Hepatology/metabolic liver disease | Fatty liver, NAFLD, MASLD, MASH, NASH, liver disease, metabolic disease | 2019 |
| Trauma/emergency/neurosurgical | Wounds, injuries, trauma, emergency care, brain injury, hemorrhage, rib fracture | 3380 |
| Obstetrics/pre-eclampsia | Pre-eclampsia, pregnancy-induced hypertension, hypertensive disorders of pregnancy | 75 |
| Oncology | Lung neoplasm, lung cancer, NSCLC, SCLC | 314 |
| Rheumatology | Rheumatoid arthritis, lupus, psoriatic arthritis, axial spondyloarthritis, vasculitis | 353 |
| Dermatology | Skin disease, psoriasis, atopic dermatitis, urticaria, acne, vitiligo, hidradenitis suppurativa, alopecia areata | 1415 |

**PubMed Search Strategies**

Hypertension

("hypertension"[MeSH Terms] OR hypertension[Title/Abstract] OR "high blood pressure"[Title/Abstract] OR "elevated blood pressure"[Title/Abstract] OR "arterial hypertension"[Title/Abstract]) AND (randomized controlled trial [Title/Abstract] OR controlled clinical trial [Title/Abstract] OR randomized [Title/Abstract] OR placebo [Title/Abstract] OR drug therapy [Title/Abstract] OR randomly [Title/Abstract] OR trial [Title/Abstract] OR groups [Title/Abstract]) AND ("2025/01/01"[Date - Publication] : "2025/12/31"[Date - Publication])

COPD

("pulmonary disease, chronic obstructive"[MeSH Terms] OR COPD[Title/Abstract] OR "chronic obstructive pulmonary disease"[Title/Abstract]) AND (randomized controlled trial [Title/Abstract] OR controlled clinical trial [Title/Abstract] OR randomized [Title/Abstract] OR placebo [Title/Abstract] OR drug therapy [Title/Abstract] OR randomly [Title/Abstract] OR trial [Title/Abstract] OR groups [Title/Abstract]) AND ("2025/01/01"[Date - Publication] : "2025/12/31"[Date - Publication])

Diabetes

("diabetes mellitus"[Title/Abstract] OR "diabete*"[Title/Abstract] OR "diabetic*"[Title/Abstract] OR "diabetic mellitus"[Title/Abstract] OR "mellitus diabetes"[Title/Abstract] OR glycuresis[Title/Abstract] OR "diabetes mellitus"[Title/Abstract] OR "blood sugar"[Title/Abstract] OR "blood glucose"[Title/Abstract] OR "HbA1C"[Title/Abstract] OR "Hemoglobin A1C"[Title/Abstract] OR "type 1 diabetes"[Title/Abstract] OR "type 2 diabetes"[Title/Abstract] OR "hyperglycemia"[Title/Abstract] OR "glycosylated hemoglobin"[Title/Abstract] OR "metabolic syndrome"[Title/Abstract] OR "T2DM"[Title/Abstract] OR "T1DM"[Title/Abstract]) AND (randomized controlled trial [Title/Abstract] OR controlled clinical trial [Title/Abstract] OR randomized [Title/Abstract] OR placebo [Title/Abstract] OR drug therapy [Title/Abstract] OR randomly [Title/Abstract] OR trial [Title/Abstract] OR groups [Title/Abstract]) AND ("2025/01/01"[Date - Publication] : "2025/12/31"[Date - Publication])

Dermatology

("Skin Diseases"[MeSH Terms] OR "Dermatology"[MeSH Terms] OR "psoriasis"[MeSH Terms] OR "Dermatitis, Atopic"[MeSH Terms] OR psoriasis[Title/Abstract] OR psoriatic[Title/Abstract] OR "atopic dermatitis"[Title/Abstract] OR eczema[Title/Abstract] OR urticaria[Title/Abstract] OR "chronic spontaneous urticaria"[Title/Abstract] OR CSU[Title/Abstract] OR acne[Title/Abstract] OR "acne vulgaris"[Title/Abstract] OR vitiligo[Title/Abstract] OR "hidradenitis suppurativa"[Title/Abstract] OR HS[Title/Abstract] OR "alopecia areata"[Title/Abstract]) AND (randomized controlled trial [Title/Abstract] OR controlled clinical trial [Title/Abstract] OR randomized [Title/Abstract] OR placebo [Title/Abstract] OR drug therapy [Title/Abstract] OR randomly [Title/Abstract] OR trial [Title/Abstract] OR groups [Title/Abstract]) AND ("2025/01/01"[Date - Publication] : "2025/12/31"[Date - Publication])

Rheumatology

("Arthritis, Rheumatoid"[MeSH Terms] OR "Spondylarthritis"[MeSH Terms] OR "Vasculitis"[MeSH Terms] OR "rheumatoid arthritis"[Title/Abstract] OR RA[Title/Abstract] OR "systemic lupus erythematosus"[Title/Abstract] OR SLE[Title/Abstract] OR lupus[Title/Abstract] OR "psoriatic arthritis"[Title/Abstract] OR PsA[Title/Abstract] OR "ankylosing spondylitis"[Title/Abstract] OR "axial spondyloarthritis"[Title/Abstract] OR axSpA[Title/Abstract] OR "giant cell arteritis"[Title/Abstract] OR GCA[Title/Abstract] OR "ANCA-associated vasculitis"[Title/Abstract] OR AAV[Title/Abstract] OR "immune-mediated inflammatory disease*"[Title/Abstract] OR IMID[Title/Abstract]) AND (randomized controlled trial [Title/Abstract] OR controlled clinical trial [Title/Abstract] OR randomized [Title/Abstract] OR placebo [Title/Abstract] OR drug therapy [Title/Abstract] OR randomly [Title/Abstract] OR trial [Title/Abstract] OR groups [Title/Abstract]) AND ("2025/01/01"[Date - Publication]: "2025/12/31"[Date - Publication])

Oncology

("Lung Neoplasms"[MeSH Terms] OR "lung cancer"[Title/Abstract] OR "lung neoplasm*"[Title/Abstract] OR "pulmonary cancer"[Title/Abstract] OR NSCLC[Title/Abstract] OR "non-small cell lung cancer"[Title/Abstract] OR "non small cell lung cancer"[Title/Abstract] OR SCLC[Title/Abstract] OR "small cell lung cancer"[Title/Abstract]) AND (randomized controlled trial [Title/Abstract] OR controlled clinical trial [Title/Abstract] OR randomized [Title/Abstract] OR placebo [Title/Abstract] OR drug therapy [Title/Abstract] OR randomly [Title/Abstract] OR trial [Title/Abstract] OR groups [Title/Abstract]) AND ("2025/01/01"[Date - Publication] : "2025/12/31"[Date - Publication])

Obstetrics/pre-eclampsia

("Pre-Eclampsia"[MeSH Terms] OR "Hypertension, Pregnancy-Induced"[MeSH Terms] OR "Pregnancy Complications, Cardiovascular"[MeSH Terms] OR "pre-eclampsia"[Title/Abstract] OR preeclampsia[Title/Abstract] OR "pre eclampsia"[Title/Abstract] OR "term pre-eclampsia"[Title/Abstract] OR "term preeclampsia"[Title/Abstract] OR "gestational hypertension"[Title/Abstract] OR "pregnancy-induced hypertension"[Title/Abstract] OR "hypertensive disorders of pregnancy"[Title/Abstract] OR "maternal hypertension"[Title/Abstract]) AND (randomized controlled trial [Title/Abstract] OR controlled clinical trial [Title/Abstract] OR randomized [Title/Abstract] OR placebo [Title/Abstract] OR drug therapy [Title/Abstract] OR randomly [Title/Abstract] OR trial [Title/Abstract] OR groups [Title/Abstract]) AND ("2025/01/01"[Date - Publication] : "2025/12/31"[Date - Publication])

Trauma/emergency/neurosurgical

("Wounds and Injuries"[MeSH Terms] OR "Multiple Trauma"[MeSH Terms] OR "Emergency Medical Services"[MeSH Terms] OR "Emergencies"[MeSH Terms] OR "Brain Injuries"[MeSH Terms] OR "Hemorrhage"[MeSH Terms] OR trauma[Title/Abstract] OR traumatic[Title/Abstract] OR injury[Title/Abstract] OR injuries[Title/Abstract] OR "multiple trauma"[Title/Abstract] OR polytrauma[Title/Abstract] OR "major trauma"[Title/Abstract] OR "severe trauma"[Title/Abstract] OR "traumatic injury"[Title/Abstract] OR "traumatic injuries"[Title/Abstract] OR "emergency care"[Title/Abstract] OR "emergency treatment"[Title/Abstract] OR "emergency department"[Title/Abstract] OR "prehospital"[Title/Abstract] OR "pre-hospital"[Title/Abstract] OR "acute care"[Title/Abstract] OR hemorrhage[Title/Abstract] OR haemorrhage[Title/Abstract] OR bleeding[Title/Abstract] OR "traumatic brain injury"[Title/Abstract] OR TBI[Title/Abstract] OR "rib fracture"[Title/Abstract] OR "rib fractures"[Title/Abstract]) AND (randomized controlled trial [Title/Abstract] OR controlled clinical trial [Title/Abstract] OR randomized [Title/Abstract] OR placebo [Title/Abstract] OR drug therapy [Title/Abstract] OR randomly [Title/Abstract] OR trial [Title/Abstract] OR groups [Title/Abstract]) AND ("2025/01/01"[Date - Publication] : "2025/12/31"[Date - Publication])

Hepatology/metabolic liver disease

("Fatty Liver"[MeSH Terms] OR "Non-alcoholic Fatty Liver Disease"[MeSH Terms] OR "Liver Diseases"[MeSH Terms] OR "Metabolic Diseases"[MeSH Terms] OR "metabolic dysfunction-associated steatotic liver disease"[Title/Abstract] OR MASLD[Title/Abstract] OR "metabolic dysfunction associated steatotic liver disease"[Title/Abstract] OR "metabolic dysfunction-associated steatohepatitis"[Title/Abstract] OR MASH[Title/Abstract] OR "non-alcoholic fatty liver disease"[Title/Abstract] OR "nonalcoholic fatty liver disease"[Title/Abstract] OR NAFLD[Title/Abstract] OR "non-alcoholic steatohepatitis"[Title/Abstract] OR "nonalcoholic steatohepatitis"[Title/Abstract] OR NASH[Title/Abstract] OR "hepatic steatosis"[Title/Abstract] OR "liver steatosis"[Title/Abstract] OR "steatotic liver disease"[Title/Abstract]) AND (randomized controlled trial [Title/Abstract] OR controlled clinical trial [Title/Abstract] OR randomized [Title/Abstract] OR placebo [Title/Abstract] OR drug therapy [Title/Abstract] OR randomly [Title/Abstract] OR trial [Title/Abstract] OR groups [Title/Abstract]) AND ("2025/01/01"[Date - Publication] : "2025/12/31"[Date - Publication])

**Identification of RCTs for Validation Dataset**

Figure S1. Screening and selection of RCTs for the external validation set

**Table S6. RCTs included in the external validation set and number of RoB records.**

| **No.** | **Clinical area** | **Title** | **DOI** | **Registry identifier** | **RoB records, n** |
| --- | --- | --- | --- | --- | --- |
| 1 | COPD | High-Flow Nasal Oxygen vs Noninvasive Ventilation in Patients With Acute Respiratory Failure: The RENOVATE Randomized Clinical Trial | 10.1001/jama.2024.26244 | NCT03643939 | 7 |
| 2 | COPD | Optimizing management beyond triple therapy in stable severe chronic obstructive pulmonary disease: efficacy of adjunctive oral doxophylline in a randomized controlled trial | 10.59556/japi.73.1207 | CTRI/2024/08/071763 | 7 |
| 3 | COPD | Telerehabilitation with breathing exercises as a strategy to improve functional capacity of patients with chronic obstructive pulmonary disease: randomized clinical trial | 10.31744/einstein_journal/2025ao1182 | Not reported in the article | 7 |
| 4 | COPD | Treating eosinophilic exacerbations of asthma and COPD with benralizumab (ABRA): a double-blind, double-dummy, active placebo-controlled randomised trial | 10.1016/s2213-2600(24)00299-6 | NCT04098718 | 6 |
| 5 | COPD | Effects of conical positive expiratory pressure mask application during exercise training on pulmonary rehabilitation outcomes in moderate to severe copd cases: a randomized controlled trial | 10.1155/pm/8828987 | Not reported in the article | 7 |
| 6 | COPD | Mepolizumab to prevent exacerbations of COPD with an eosinophilic phenotype | 10.1056/nejmoa2413181 | NCT04133909 | 7 |
| 7 | Diabetes | A randomized trial of automated insulin delivery in type 2 diabetes | 10.1056/nejmoa2415948 | NCT05785832 | 7 |
| 8 | Diabetes | Effect of ketogenic diet plus dulaglutide on glucose and lipid metabolism in diabetes mellitus | 10.36721/pjps.2025.38.6.reg.13419.1 | Not reported in the article | 7 |
| 9 | Diabetes | Effectiveness of regular oat beta-glucan-enriched bread compared with whole-grain wheat bread on long-term glycemic control in adults at risk of type 2 diabetes: a randomized controlled trial | 10.1016/j.ajcnut.2025.06.018 | NCT04994327 | 7 |
| 10 | Diabetes | Subcutaneous weekly semaglutide with automated insulin delivery in type 1 diabetes: a double-blind, randomized, crossover trial | 10.1038/s41591-024-03463-z | NCT05205928 | 7 |
| 11 | Diabetes | Dual therapy triumph: Yuquan capsules and metformin in combating type 2 diabetes mellitus disorders | 10.1002/cpdd.1592 | Not reported in the article | 6 |
| 12 | Diabetes | Orforglipron, an oral small-molecule GLP-1 receptor agonist, for the treatment of obesity in people with type 2 diabetes (ATTAIN-2): a phase 3, double-blind, randomised, multicentre, placebo-controlled trial | 10.1016/s0140-6736(25)02165-8 | NCT05872620 | 6 |
| 13 | Hypertension | Add-on treatment with Zilebesiran for inadequately controlled hypertension: The KARDIA-2 randomized clinical trial | 10.1001/jama.2025.6681 | NCT05103332 | 6 |
| 14 | Hypertension | Efficacy and safety of allisartan isoproxil/amlodipine in patients with essential hypertension: A phase III, multicenter, double-blind, parallel-group, randomised study | 10.1038/s41371-025-01035-3 | NCT06465264 | 7 |
| 15 | Hypertension | Preliminary, randomized, double-blinded, placebo-controlled cross-over study with resveratrol in hypertensive patients | 10.1038/s41598-025-17305-6 | IRCT201407078129N7 | 7 |
| 16 | Hypertension | Spironolactone vs Amiloride for resistant hypertension: a randomized clinical trial | 10.1001/jama.2025.5129 | NCT04331691 | 7 |
| 17 | Hypertension | Heart rate variability and blood pressure response to low-intensity endurance exercise training plus blood flow restriction in individuals with mild hypertension: a randomized controlled clinical trial | 10.30476/ijms.2025.105405.3916 | IRCT20230528058311N1 | 7 |
| 18 | Hypertension | Baxdrostat efficacy and safety in uncontrolled and resistant hypertension | 10.1056/nejmoa2507109 | NCT06034743 | 6 |
| 19 | Dermatology | Efficacy and safety of cannabidiol oil in psoriasis: a randomized, double-blind, placebo-controlled trial | 10.1080/09546634.2025.2604448 | TCTR20220622002 | 7 |
| 20 | Hepatology/metabolic liver disease | Tenofovir alafenamide for prevention of HBV reactivation in HBsAg-negative, anti-HBc-positive patients undergoing rituximab-based chemotherapy: A multicenter randomized controlled trial | 10.1097/hc9.0000000000000859 | NCT02186574 | 6 |
| 21 | Obstetrics/pre-eclampsia | Scheduled birth at term for the prevention of pre-eclampsia (PREVENT-PE): an open-label randomised controlled trial | 10.1016/s0140-6736(25)01207-3 | ISRCTN41632964 | 6 |
| 22 | Oncology | A multicenter randomized controlled trial of intrapleural perfusion of methotrexate-loaded tumor cell-derived microparticles combined with systemic therapy for malignant pleural effusion | 10.1002/ijc.70094 | ChiCTR1800017104 | 7 |
| 23 | Rheumatology | Efficacy and safety of iguratimod in patients with primary Sjogren's syndrome: a multicentre randomised controlled trial | 10.1136/rmdopen-2025-006180 | NCT04981145 | 7 |
| 24 | Trauma/emergency/neurosurgical | Dural tenting in elective craniotomies: a randomized clinical trial | 10.1227/neu.0000000000003480 | NCT03658941 | 7 |

Notes: Each RCT contributed one record for each of the RoB 1 domains: random sequence generation, allocation concealment, blinding of participants and personnel, incomplete outcome data, and other bias. Blinding of outcome assessment was assessed separately for each applicable outcome type, yielding one additional record per outcome type.
