# Supplementary material for: A Fine-Tuned Large Language Model Workflow to Support Risk of Bias Assessment: Development, External Validation, and Reviewer Verification Study
Source: J Med Internet Res. 2026 Nov 13;28:e108001. doi: 10.2196/108001 (PMC13765787; doi:10.2196/108001)
Supplement: Supplementary file 5 [file jmir-v28-e108001-s003.docx]

**Rule-Based Generation and Distribution of Candidate Evidence Hints**

**Generation Procedure**

Candidate evidence hints were generated automatically after the clinician specified the RoB 1 domain and selected the source passage. Matching was deterministic and domain-specific, not based on an additional LLM. Before matching, domain labels were standardized by removing bias types in parentheses, e.g., “(selection bias)” from “Random sequence generation (selection bias)”.

After replacing repeated spaces and line breaks with single spaces, the passage was split at sentence-ending punctuation and at numbered subsection headings. If fewer than three segments were produced, semicolons and colons were also used. Matching was case-insensitive.

Each sentence was assigned a score using the domain-specific rules below. Each matched indicator contributed its score once. Sentences longer than 45 words received a 1-point penalty, and those longer than 70 words received an additional 2-point penalty. Sentences scoring >0 were ranked by score, with equal-scoring sentences retained their original order. Case-insensitive identical duplicates were removed, and up to three sentences were returned as candidate evidence hints. If no sentence scored positively, no candidate evidence hints were generated.

**Table S7. Domain-specific positive scoring rules**

| **RoB 1 domains** | **Positive indicators and scores** |
| --- | --- |
| Random sequence generation | computer-generated or random number (+8); block or stratified block randomization (+7); randomization/randomisation or randomly assigned/allocated (+6); interactive web-response system, IWRS, or IVRS (+5); sequence (+3); stratified or assigned in a specified ratio (+2); group (+1) |
| Allocation concealment | opaque sealed envelope(s) (+10); sealed or numbered envelopes, central randomization/allocation, allocation concealment, or concealed (+8); sequentially numbered, IWRS, or IVRS (+6); enrolment handled separately from group assignment (+5); assignment and sealed (+4); allocation or envelope (+2); group or participant(s) (+1) |
| Blinding of participants and personnel | remained/were masked or masked to the intervention (+10); single-, double-, or triple-blind, participant/personnel/investigator blinded, or open-label (+9); placebo, sham, or without a device (+7); group using a device or control without an intervention/device (+6); used a device (+5); control group, blinded, or masked (+4); intervention (+3); site staff or investigator(s) (+2); participant(s), personnel, or group (+1) |
| Blinding of outcome assessment | blinded outcome assessor(s) (+10); masked or independent assessor (+9); outcome assessment (+7); objective or subjective (+6); assessed/measured using, questionnaire, CAT, SGRQ, TDI, or BDI (+4); exercise capacity, dyspnea, HRQoL, quality of life, 6-minute walk test, 6MWT, ESMT, spirometer, or pedometer (+3); assessed, measured, or outcome (+2); baseline, end of the program, or follow-up (+1) |
| Incomplete outcome data | intention-to-treat/ITT, imputation, lost to follow-up, missing data, or attrition (+8); dropout, withdrawal, or discontinuation (+7); completed the study/follow-up or sensitivity analysis (+4); per-protocol (+3); analysis (+2); P value (+1); STATA, SPSS, or software (0) |
| Other bias | baseline imbalance or early stopping (+9); protocol deviation, carryover, or contamination (+8); conflict of interest (+7); funding or sponsor (+5); disclosure, industry, run-in, or cluster (+3); baseline (+2) |

**Table S8. Domain-specific negative scoring rules**

| **RoB 1 domains** | **Negative indicators and scores** |
| --- | --- |
| Random sequence generation | informed consent, recruitment, eligibility, or baseline characteristics (−4) |
| Allocation concealment | randomization alone (−1); informed consent, recruitment, or eligibility (−4) |
| Blinding of participants and personnel | informed consent (−8); recruitment or eligibility criteria (−6); ethics approval or registration statements (−5); hospital (−2) |
| Blinding of outcome assessment | informed consent, recruitment, eligibility, or ethics committee (−5) |
| Incomplete outcome data | general statistical-method terms, including descriptive statistics, ANOVA, Pearson, Shapiro, Wilcoxon, Mann-Whitney, chi2, and ANCOVA (−4); baseline characteristics (−2) |
| Other bias | informed consent, recruitment, or eligibility (−5) |

**Table S9.** **Distribution of candidate evidence hints generated from clinician-selected passages**

| **Number of candidate hints** | **From Clinician 1, n** | **From Clinician 2, n** |
| --- | --- | --- |
| 0 | 16 | 28 |
| 1 | 23 | 40 |
| 2 | 33 | 38 |
| 3 | 89 | 55 |
| Passages with ≥1 hint | 145 (90.1%) | 133 (82.6%) |
| Total hints | 356 | 281 |

**Table S10. Distribution of candidate evidence hints by RoB 1 domains and clinicians**

| **RoB 1 domains** | **Records, n** | **Clinician 1** | | **Clinician 2** | |
| --- | --- | --- | --- | --- | --- |
|  |  | **Records with ≥1 hint** | **Total hints** | **Records with ≥1 hint** | **Total hints** |
| Random sequence generation | 24 | 24 (100.0%) | 60 | 24 (100.0%) | 57 |
| Allocation concealment | 24 | 22 (91.7%) | 51 | 21 (87.5%) | 43 |
| Blinding of participants and personnel | 24 | 24 (100.0%) | 64 | 24 (100.0%) | 54 |
| Blinding of outcome assessment | 41 | 40 (97.6%) | 107 | 34 (82.9%) | 75 |
| Incomplete outcome data | 24 | 20 (83.3%) | 51 | 18 (75.0%) | 35 |
| Other bias | 24 | 15 (62.5%) | 23 | 12 (50.0%) | 17 |
| Total | 161 | 145 (90.1%) | 356 | 133 (82.6%) | 281 |
