# Supplementary material for: A Fine-Tuned Large Language Model Workflow to Support Risk of Bias Assessment: Development, External Validation, and Reviewer Verification Study
Source: J Med Internet Res. 2026 Nov 13;28:e108001. doi: 10.2196/108001 (PMC13765787; doi:10.2196/108001)
Supplement: Supplementary file 6 [file jmir-v28-e108001-s004.docx]

**Domain-Specific RoB Assessment Rules**

**Table S11. Domain-specific RoB assessment rules**

| **RoB 1 domain** | **Rule summary** |
| --- | --- |
| **Random sequence generation** | **Low risk:** Explicit use of a truly random method, such as a random number table, computer-generated random numbers, or a random number generator.  **High risk:** Nonrandom methods, such as alternation, date of birth, admission number, record number, or allocation by date.  **Unclear risk:** The study was described as randomized, but the method of sequence generation was not reported in sufficient detail. |
| **Allocation concealment** | **Low risk:** Allocation was adequately concealed, such as through central randomization, pharmacy-controlled allocation, or sequentially numbered, opaque, sealed envelopes.  **High risk:** Allocation was not concealed or could have been foreseen, such as through open allocation lists or transparent or unsealed envelopes.  **Unclear risk:** Insufficient detail was available to determine whether allocation concealment was adequate. |
| **Blinding of participants and personnel** | **Low risk:** Participants and study personnel were blinded, or lack of blinding was unlikely to influence the outcome.  **High risk:** Blinding was absent or incomplete, and the outcome was likely to be influenced by lack of blinding.  **Unclear risk:** Insufficient information was available about who was blinded or whether lack of blinding could have affected the outcome. |
| **Blinding of outcome assessment** | **Low risk:** Outcome assessors were blinded, or the outcome was objective and unlikely to be influenced by lack of blinding.  **High risk:** Outcome assessors were not blinded, and the outcome was subjective or likely to be influenced by knowledge of the intervention.  **Unclear risk:** Insufficient information was available to determine whether blinding of outcome assessment was adequate. |
| **Incomplete outcome data** | **Low risk:** Missing outcome data were minimal, balanced across groups, or adequately handled.  **High risk:** There were substantial missing data, imbalance between groups, reasons for missingness related to outcomes, or inappropriate handling of missing data.  **Unclear risk:** Insufficient information was available about the amount, reasons, or handling of missing outcome data. |
| **Other bias** | **Low risk:** No important additional source of bias was identified.  **High risk:** A clear additional source of bias was present, such as a major baseline imbalance, early stopping, problematic design features, or sponsor-related concerns that affected the study.  **Unclear risk:** Another source of bias may have been present, but the available information was insufficient to determine its effect. |

**References**

Higgins JPT, Altman DG. Assessing risk of bias in included studies. In: Higgins JPT, Green S, editors. Cochrane Handbook for Systematic Reviews of Interventions. Wiley-Blackwell; 2008:187-241. doi:10.1002/9780470712184.ch8.
