# Supplementary material for: A Fine-Tuned Large Language Model Workflow to Support Risk of Bias Assessment: Development, External Validation, and Reviewer Verification Study
Source: J Med Internet Res. 2026 Nov 13;28:e108001. doi: 10.2196/108001 (PMC13765787; doi:10.2196/108001)
Supplement: Supplementary file 8 [file jmir-v28-e108001-s006.docx]

**Inference Settings and** **Output Checks**

**Inference Settings**

**Table S12. Model loading and decoding settings**

| **Settings** | **Value** |
| --- | --- |
| Base model | Gemma 2-9B-Instruct |
| Adapter | Retained stage 2 adapter |
| Model loading | 4-bit bitsandbytes; NF4; double quantization |
| Compute type | float16 |
| Temperature | 0.0 |
| Sampling | Disabled |
| Repetition penalty | 1.0 |
| Maximum new tokens | Step 1: 1024; retry: 220; fallback: 180; Step 2: 192 |

**Output Checks**

**Table S13 Predefined output checks**

| **Check** | **Operational rules** |
| --- | --- |
| JSON parsing | The response had to contain one valid JSON object. |
| Required fields | bias_domain, judgement, and supporting_quote had to be present. |
| Judgement category | The accepted categories were Low risk, Unclear risk, and High risk. |
| Quotation traceability | Quotations had to match a continuous span in the reviewer-supplied text. White-space and common Unicode variants were normalized when needed. |
| Retry | Both workflows followed the same retry procedure. An invalid first-pass output received one retry using a shorter corrective prompt. |
| Fallback | If the retry remained invalid, one fallback generation used concatenated candidate evidence hints (up to 1800 characters). When no hints were available, the first 1800 characters of the Step 1 input excerpt were used. The fallback used a dedicated structured-output prompt and a maximum of 180 new tokens. Each record received at most two retries, including the fallback attempt. |

**Table S14. Output validity on the first-pass and retry outcomes**

| **Model** | **Invalid first outputs, n/N (%)** | **Records requiring one retry, n** | **Records requiring two retries, n** | **Records with changed judgments, n** |
| --- | --- | --- | --- | --- |
| **Clinician 1** | | | | |
| Gemma 2 base model | 3/161 (1.9%) | 0 | 3 | 1 |
| TRoB-1 | 2/161 (1.2%) | 1 | 1 | 0 |
| **Clinician 2** | | | | |
| Gemma 2 base model | 26/161 (16.1%) | 4 | 22 | 5 |
| TRoB-1 | 2/161 (1.2%) | 2 | 0 | 0 |
