# Supplementary material for: A Fine-Tuned Large Language Model Workflow to Support Risk of Bias Assessment: Development, External Validation, and Reviewer Verification Study
Source: J Med Internet Res. 2026 Nov 13;28:e108001. doi: 10.2196/108001 (PMC13765787; doi:10.2196/108001)
Supplement: Supplementary file 9 [file jmir-v28-e108001-s007.docx]

**Statistical Analysis Software and Package Versions**

**Table S15 Versions of software or packages**

| **Purpose** | **Software or packages** | **Version** |
| --- | --- | --- |
| Programming language | Python | 3.11.6 |
| Numerical operations | NumPy | 2.3.4 |
| Data management | pandas | 2.2.3 |
| Performance metrics | scikit-learn | 1.7.2 |
| Statistical tests | SciPy | 1.16.2 |
| Spreadsheet input and output | openpyxl | 3.1.5 |
