# Supplementary material for: A Fine-Tuned Large Language Model Workflow to Support Risk of Bias Assessment: Development, External Validation, and Reviewer Verification Study
Source: J Med Internet Res. 2026 Nov 13;28:e108001. doi: 10.2196/108001 (PMC13765787; doi:10.2196/108001)
Supplement: Supplementary file 10 [file jmir-v28-e108001-s008.docx]

**Performance Comparisons and Sensitivity Analyses**

**Table S16 Sensitivity analysis excluding invalid base-model outputs**

| **Clinician** | **Valid records, n** | **Gemma 2 base accuracy, % (95% CI)** | **TRoB-1 raw accuracy, % (95% CI)** | **P value** |
| --- | --- | --- | --- | --- |
| Clinician 1 | 158 | 61.4 (53.9–68.7) | 74.1 (68.6–79.5) | .004 |
| Clinician 2 | 136 | 61.8 (53.2–70.8) | 72.8 (66.4–79.3) | .038 |

Notes: Records with invalid Gemma 2 base outputs were excluded, leaving 158 and 136 records for Clinicians 1 and 2, respectively. For each clinician, both workflows were evaluated on the same retained subset.

**Table S17 Paired performance differences between TRoB-1 raw outputs and the Gemma 2 base workflow**

| **Metric** | **Differences** | **95% CI** | **P value** |
| --- | --- | --- | --- |
| **Clinician 1** | | | |
| Accuracy | 13.7% | 5.7%–22.2% | .004 |
| Macro-F1 | 0.086 | 0.007–0.161 | — |
| MCC | 0.136 | 0.015–0.255 | — |
| **Clinician 2** | | | |
| Accuracy | 21.1% | 11.7%–30.4% | <.001 |
| Macro-F1 | 0.109 | 0.015–0.201 | — |
| MCC | 0.180 | 0.062–0.292 | — |

Notes: All 161 records for each clinician were included, with invalid Gemma 2 base outputs counted as incorrect. Differences were calculated as between TRoB-1 raw outputs and the Gemma 2 base workflow.

**Table S18. Trial-level sensitivity analysis for the two independent clinician assessments (24 RCTs each)**

| **Clinician-assisted run** | **Model** | **Mean trial-level accuracy (95% CI)** | **Comparison** | **Difference, (95% CI)** | | **P value** |
| --- | --- | --- | --- | --- | --- | --- |
| Clinician 1 | Gemma 2 base | 60.7% (52.1%-68.6%) | NA | NA | NA | |
| Clinician 1 | TRoB-1 raw | 74.4% (68.8%-79.8%) | TRoB-1 raw minus Gemma 2 base | 13.7% (6.0%-21.9%) | .002 | |
| Clinician 1 | Clinician-verified TRoB-1 | 82.2% (76.0%-88.1%) | Clinician-verified minus TRoB-1 raw | 7.8% (4.2%-11.6%) | <.001 | |
| Clinician 2 | Gemma 2 base | 52.9% (44.0%-62.1%) | NA | NA | NA | |
| Clinician 2 | TRoB-1 raw | 73.8% (67.4%-80.3%) | TRoB-1 raw minus Gemma 2 base | 20.9% (11.5%-30.3%) | <.001 | |
| Clinician 2 | Clinician-verified TRoB-1 | 84.8% (79.7%-89.8%) | Clinician-verified minus TRoB-1 raw | 11.0 %(6.7%-15.4%) | <.001 | |

Notes: Accuracy was calculated per RCT and then averaged across all 24 RCTs.
