# Supplementary material for: A Fine-Tuned Large Language Model Workflow to Support Risk of Bias Assessment: Development, External Validation, and Reviewer Verification Study
Source: J Med Internet Res. 2026 Nov 13;28:e108001. doi: 10.2196/108001 (PMC13765787; doi:10.2196/108001)
Supplement: Supplementary file 11 [file jmir-v28-e108001-s009.docx]

Case Level Analysis of Judgment Discrepancies in the Two Clinician-Assisted Runs

**Table S19. Judgments not matching the reference standard by RoB 1 domain**

| **RoB 1 domains** | **Records, n** | **Clinician 1 TRoB-1 raw, n/N (%)** | **Clinician 1 verified, n/N (%)** | **Clinician 2 TRoB-1 raw, n/N (%)** | **Clinician 2 verified, n/N (%)** |
| --- | --- | --- | --- | --- | --- |
| Random sequence generation | 24 | 2/24 (8.3) | 2/24 (8.3) | 1/24 (4.2) | 1/24 (4.2) |
| Allocation concealment | 24 | 6/24 (25.0) | 6/24 (25.0) | 8/24 (33.3) | 5/24 (20.8) |
| Blinding of participants and personnel | 24 | 5/24 (20.8) | 3/24 (12.5) | 4/24 (16.7) | 1/24 (4.2) |
| Blinding of outcome assessment | 41 | 12/41 (29.3) | 5/41 (12.2) | 11/41 (26.8) | 7/41 (17.1) |
| Incomplete outcome data | 24 | 9/24 (37.5) | 8/24 (33.3) | 8/24 (33.3) | 3/24 (12.5) |
| Other bias | 24 | 8/24 (33.3) | 5/24 (20.8) | 11/24 (45.8) | 8/24 (33.3) |
| Total | 161 | 42/161 (26.1) | 29/161 (18.0) | 43/161 (26.7) | 25/161 (15.5) |

Notes: Values are the numbers and percentages of nonmatching judgments within each domain.

**Table S20. Directions of judgments not matching the reference standard**

| **Reference-standard judgment** | **Workflow judgement** | **Clinician 1 TRoB-1 raw, n** | **Clinician 1 verified, n** | **Clinician 2 TRoB-1 raw, n** | **Clinician 2 verified, n** |
| --- | --- | --- | --- | --- | --- |
| Low risk | Unclear risk | 8 | 5 | 8 | 7 |
| Low risk | High risk | 7 | 3 | 10 | 5 |
| Unclear risk | Low risk | 14 | 13 | 11 | 8 |
| Unclear risk | High risk | 1 | 1 | 3 | 1 |
| High risk | Low risk | 1 | 0 | 2 | 0 |
| High risk | Unclear risk | 11 | 7 | 9 | 4 |
| **Total, n** | | 42 | 29 | 43 | 25 |

**Table S21.** **Deidentified case-level summary of assessment targets with judgments not matching the reference standard**

| **Assessment ID** | **RoB 1 domains** | **Outcome type** | **Reference judgment** | **Clinician 1 TRoB-1 raw / verified** | **Clinician 2 TRoB-1 raw / verified** |
| --- | --- | --- | --- | --- | --- |
| A003 | Blinding of participants and personnel | All outcomes | High | High / High | Unclear / High |
| A004 | Blinding of outcome assessment | Subjective outcomes | High | Unclear / High | Unclear / High |
| A005 | Blinding of outcome assessment | Objective outcomes | Low | Low / Low | Unclear / Unclear |
| A015 | Allocation concealment | NA | Low | Low / Low | Unclear / Unclear |
| A016 | Blinding of participants and personnel | All outcomes | High | Low / High | Unclear / Unclear |
| A022 | Allocation concealment | NA | Unclear | Low / Low | Low / Low |
| A024 | Blinding of outcome assessment | Subjective outcomes | Unclear | Low / Unclear | Low / Low |
| A026 | Incomplete outcome data | All outcomes | Low | High / Low | High / Low |
| A028 | Random sequence generation | NA | Unclear | Low / Low | Low / Low |
| A029 | Allocation concealment | NA | Unclear | Unclear / Unclear | Low / Low |
| A031 | Blinding of outcome assessment | Subjective outcomes | High | Unclear / Unclear | Unclear / High |
| A034 | Other bias | NA | High | Unclear / Unclear | High / High |
| A036 | Allocation concealment | NA | Unclear | Low / Low | Low / Low |
| A038 | Blinding of outcome assessment | Subjective outcomes | Unclear | Unclear / Unclear | Low / Low |
| A040 | Incomplete outcome data | All outcomes | Unclear | Low / Low | High / High |
| A047 | Incomplete outcome data | All outcomes | Low | Low / Low | High / High |
| A048 | Other bias | NA | Low | Unclear / Unclear | Low / Low |
| A049 | Allocation concealment | NA | Unclear | Low / Low | Low / Unclear |
| A050 | Blinding of outcome assessment | Subjective outcomes | High | Unclear / Unclear | Unclear / Unclear |
| A051 | Blinding of outcome assessment | Objective outcomes | Unclear | Low / Low | Low / Low |
| A053 | Incomplete outcome data | All outcomes | Unclear | High / High | Unclear / Unclear |
| A057 | Allocation concealment | NA | Unclear | Low / Low | Low / Low |
| A059 | Blinding of outcome assessment | Subjective outcomes | Low | High / Low | Low / Low |
| A062 | Other bias | NA | Low | Unclear / Unclear | Unclear / Low |
| A065 | Blinding of participants and personnel | All outcomes | High | Unclear / High | High / High |
| A066 | Blinding of outcome assessment | Subjective outcomes | High | Unclear / High | Unclear / High |
| A069 | Other bias | NA | Low | Low / Low | Unclear / Low |
| A073 | Blinding of outcome assessment | Subjective outcomes | Low | Unclear / Low | Low / Low |
| A075 | Incomplete outcome data | All outcomes | High | High / High | Unclear / High |
| A076 | Other bias | NA | Low | Unclear / Unclear | Low / Low |
| A078 | Allocation concealment | NA | Unclear | Low / Low | High / Unclear |
| A080 | Blinding of outcome assessment | Subjective outcomes | Low | Unclear / Low | Low / Low |
| A082 | Incomplete outcome data | All outcomes | Low | High / High | Low / Low |
| A085 | Allocation concealment | NA | Unclear | Low / Low | Low / Unclear |
| A088 | Incomplete outcome data | All outcomes | Unclear | Low / Low | Low / Unclear |
| A089 | Other bias | NA | High | High / High | Unclear / Unclear |
| A098 | Blinding of participants and personnel | All outcomes | High | Unclear / Unclear | Low / High |
| A102 | Other bias | NA | Low | Unclear / Low | High / High |
| A109 | Other bias | NA | Low | Unclear / Low | High / High |
| A110 | Random sequence generation | NA | Unclear | Low / Low | Unclear / Unclear |
| A112 | Blinding of participants and personnel | All outcomes | High | Unclear / Unclear | Low / High |
| A113 | Blinding of outcome assessment | Subjective outcomes | Unclear | Unclear / Unclear | High / Unclear |
| A115 | Incomplete outcome data | All outcomes | Low | Low / Low | High / High |
| A116 | Other bias | NA | Low | Low / Low | High / High |
| A121 | Incomplete outcome data | All outcomes | Unclear | Low / Low | Unclear / Unclear |
| A122 | Other bias | NA | Low | Low / Low | Unclear / Unclear |
| A128 | Other bias | NA | Low | High / Low | High / Low |
| A134 | Other bias | NA | Low | High / Unclear | High / Unclear |
| A139 | Blinding of outcome assessment | Objective outcomes | Unclear | Low / Low | Unclear / Unclear |
| A140 | Incomplete outcome data | All outcomes | Low | High / High | High / Low |
| A146 | Blinding of outcome assessment | Objective outcomes | Low | Unclear / Unclear | Unclear / Unclear |
| A147 | Incomplete outcome data | All outcomes | Low | High / High | High / Low |
| A148 | Other bias | NA | Low | Low / Low | Unclear / Unclear |
| A152 | Blinding of outcome assessment | Subjective outcomes | High | Unclear / High | Unclear / Unclear |
| A154 | Incomplete outcome data | All outcomes | High | Unclear / Unclear | High / High |
| A155 | Other bias | NA | Low | Low / Low | Unclear / Unclear |
| A158 | Blinding of participants and personnel | All outcomes | High | Unclear / Unclear | High / High |

Notes: The case-level summary includes the 57 assessment records for which at least one judgment (raw or verified, from either clinician) does not match the reference standard. Assessment IDs have been deidentified and follow the reference order. NA: not applicable.
