# Supplementary material for: A Fine-Tuned Large Language Model Workflow to Support Risk of Bias Assessment: Development, External Validation, and Reviewer Verification Study
Source: J Med Internet Res. 2026 Nov 13;28:e108001. doi: 10.2196/108001 (PMC13765787; doi:10.2196/108001)
Supplement: Supplementary file 12 [file jmir-v28-e108001-s010.docx]

Subgroup Performance of TRoB-1 and Agreement Between Independent Clinicians

**Table S22. Accuracy by clinical areas**

| **Clinical areas** | **Clinicians** | **Records, n** | **Gemma 2 base accuracy (95% CI)** | **TRoB-1 raw accuracy (95% CI)** | **Clinician-verified accuracy (95% CI)** |
| --- | --- | --- | --- | --- | --- |
| COPD | Clinician 1 | 41 | 0.683 (0.619-0.750) | 0.732 (0.571-0.875) | 0.805 (0.634-0.949) |
|  | Clinician 2 | 41 | 0.634 (0.524-0.775) | 0.805 (0.690-0.900) | 0.878 (0.805-0.951) |
| Diabetes | Clinician 1 | 40 | 0.625 (0.513-0.732) | 0.750 (0.667-0.825) | 0.825 (0.700-0.949) |
|  | Clinician 2 | 40 | 0.550 (0.366-0.744) | 0.700 (0.610-0.800) | 0.825 (0.700-0.949) |
| Hypertension | Clinician 1 | 40 | 0.500 (0.286-0.684) | 0.725 (0.619-0.842) | 0.850 (0.756-0.949) |
|  | Clinician 2 | 40 | 0.475 (0.282-0.700) | 0.700 (0.561-0.842) | 0.850 (0.756-0.949) |
| Out-of-domain | Clinician 1 | 40 | 0.600 (0.415-0.805) | 0.750 (0.700-0.805) | 0.800 (0.718-0.900) |
|  | Clinician 2 | 40 | 0.425 (0.333-0.526) | 0.725 (0.600-0.850) | 0.825 (0.732-0.925) |

Notes: Accuracy estimates were calculated separately for each clinician-assisted assessment. Invalid or incomplete Gemma 2 base outputs were counted as incorrect. The 95% CIs were estimated using 50,000 RCT-cluster bootstrap replicates. COPD: chronic obstructive pulmonary disease.

**Table S23.** **Accuracy by RoB 1 domains**

| **RoB 1 domains** | **Clinicians** | **Records, n** | **Gemma 2 base accuracy (95% CI)** | **TRoB-1 raw accuracy (95% CI)** | **Clinician-verified accuracy (95% CI)** |
| --- | --- | --- | --- | --- | --- |
| Random sequence generation | Clinician 1 | 24 | 0.792 (0.625-0.958) | 0.917 (0.792-1.000) | 0.917 (0.792-1.000) |
|  | Clinician 2 | 24 | 0.542 (0.333-0.750) | 0.958 (0.875-1.000) | 0.958 (0.875-1.000) |
| Allocation concealment | Clinician 1 | 24 | 0.625 (0.417-0.792) | 0.750 (0.583-0.917) | 0.750 (0.583-0.917) |
|  | Clinician 2 | 24 | 0.583 (0.375-0.792) | 0.667 (0.458-0.833) | 0.792 (0.625-0.958) |
| Blinding of participants and personnel | Clinician 1 | 24 | 0.708 (0.500-0.875) | 0.792 (0.625-0.958) | 0.875 (0.750-1.000) |
|  | Clinician 2 | 24 | 0.583 (0.375-0.792) | 0.833 (0.667-0.958) | 0.958 (0.875-1.000) |
| Blinding of outcome assessment | Clinician 1 | 41 | 0.390 (0.233-0.556) | 0.707 (0.587-0.829) | 0.878 (0.756-0.976) |
|  | Clinician 2 | 41 | 0.317 (0.184-0.462) | 0.732 (0.590-0.872) | 0.829 (0.703-0.946) |
| Incomplete outcome data | Clinician 1 | 24 | 0.667 (0.458-0.833) | 0.625 (0.417-0.792) | 0.667 (0.458-0.833) |
|  | Clinician 2 | 24 | 0.750 (0.583-0.917) | 0.667 (0.458-0.833) | 0.875 (0.750-1.000) |
| Other bias | Clinician 1 | 24 | 0.583 (0.375-0.792) | 0.667 (0.458-0.833) | 0.792 (0.625-0.958) |
|  | Clinician 2 | 24 | 0.500 (0.292-0.708) | 0.542 (0.333-0.750) | 0.667 (0.458-0.833) |

Notes: Accuracy estimates were calculated separately for each clinician-assisted assessment. Invalid or incomplete Gemma 2 base outputs were counted as incorrect. The 95% CIs were estimated using 50,000 RCT-cluster bootstrap replicates. RoB 1: Cochrane risk-of-bias tool version 1.

**Table S24. Agreement between two clinicians on final RoB 1 judgments**

| **Final judgment of Clinician 1** | **Final judgment of Clinician 2** | | | **Total, n** |
| --- | --- | --- | --- | --- |
|  | **Low risk** | **Unclear risk** | **High risk** |  |
| **Low risk** | 97 | 12 | 6 | 115 |
| **Unclear risk** | 6 | 10 | 6 | 22 |
| **High risk** | 3 | 4 | 17 | 24 |
| **Total, n** | 106 | 26 | 29 | 161 |

Notes: Values are numbers of assessment records. Overall agreement was 124/161 (77.0%; 95% CI 70.8%-83.1%), Gwet's AC1 was 0.698 (95% CI 0.601-0.788).
