# Supplementary material for: A Fine-Tuned Large Language Model Workflow to Support Risk of Bias Assessment: Development, External Validation, and Reviewer Verification Study
Source: J Med Internet Res. 2026 Nov 13;28:e108001. doi: 10.2196/108001 (PMC13765787; doi:10.2196/108001)
Supplement: Supplementary file 13 [file jmir-v28-e108001-s012.docx]

Reproducibility Across Runs and Hardware Environments

Reproducibility was assessed using the 322 assessment inputs from the two clinician-assisted runs (161 per run). The complete TRoB-1 workflow was repeated on a laptop and compared with the original source-server outputs generated from the same inputs, model adapter, prompt templates, candidate-hint procedure, output checks, and deterministic decoding settings. A second Step 1 run was performed on the laptop to assess within-hardware repeatability.

**Table S25. Hardware and software environments used for the reproducibility assessment**

| **Component** | **Original source server** | **Laptop reproducibility run** |
| --- | --- | --- |
| Operating system | Ubuntu 22.04 | Windows 11 (build 26200) |
| Graphics processor | NVIDIA GeForce RTX 4090 | NVIDIA GeForce RTX 5070 Ti Laptop GPU |
| Graphics memory | 24 GB | 12,226 MiB |
| Python | 3.11 | 3.11.15 |
| PyTorch | 2.10.0+cu128 | 2.8.0+cu128 |
| CUDA | 12.8 | 12.8 |
| Transformers | 4.57.6 | 5.9.0 |
| PEFT | 0.18.1 | 0.19.1 |
| bitsandbytes | 0.49.2 | 0.49.2 |
| Quantization | 4-bit NF4 with double quantization | 4-bit NF4 with double quantization |
| Compute type | float16 | float16 |
| Temperature | 0.0 | 0.0 |
| Sampling | Disabled | Disabled |

Notes: The original source-server environment corresponds to the environment used for the original TRoB-1 inference runs. The same base model, retained stage 2 adapter, prompt templates, output checks, retry procedure, and decoding settings were used in both environments. Output validity required parseable JSON with the required fields, an accepted RoB 1 judgment, and a traceable supporting quotation. Judgment agreement required identical RoB 1 categories; quotation agreement required identical text after whitespace and soft-hyphen normalization; and exact Step 1 agreement required character-for-character identity. Source-server validity was assessed after the predefined retries.

**Table S26. Reproducibility results across runs and hardware environments**

| **Comparison and outcome** | **Clinician 1** | **Clinician 2** | **Overall** |
| --- | --- | --- | --- |
| Valid output, original source server | 161/161 (100%) | 161/161 (100%) | 322/322 (100%) |
| Valid output, laptop complete workflow | 160/161 (99.4%) | 161/161 (100%) | 321/322 (99.7%) |
| Same RoB 1 judgment, server vs laptop | 160/161 (99.4%) | 159/161 (98.8%) | 319/322 (99.1%) |
| Same supporting quotation, server vs laptop | 156/161 (96.9%) | 158/161 (98.1%) | 314/322 (97.5%) |
| Exact Step 1 response, server vs laptop | 155/161 (96.3%) | 156/161 (96.9%) | 311/322 (96.6%) |
| Exact Step 1 response, repeated laptop runs | 161/161 (100%) | 161/161 (100%) | 322/322 (100%) |

Notes: Values are n/N (%). Complete-workflow validity covered candidate-hint generation, Step 1 checks and retries, quotation traceability, and Step 2. Judgment and quotation agreement were assessed per input; exact-response agreement used the final Step 1 response string. Exact text agreement for the Step 2 explanations was 225/322 (69.9%). Since natural-language explanations may vary in wording, this result was not used as the main measure of reproducibility.
